# Cyclodextrin inclusion complexes enhance the solubility and anti-virulence activity of metronidazole against uropathogenic *Proteus mirabilis*

**DOI:** 10.1371/journal.pone.0353058

**Published:** 2026-07-27

**Authors:** Rehab Mahmoud Abd El-Baky, Zeinab Fathalla, Hala Rady Ahmed, Ramadan Yahia, Mohamed A. Mawhoup, Ghada M. Sadiq, Hebatallah S. Barakat, Adel Al Fatease, Ali H. Alamri, Hamdy Abdelkader

**Affiliations:** 1 Department of Microbiology and Immunology, Faculty of Pharmacy, Minia University, Minia, Egypt; 2 Department of Microbiology and Immunology, Faculty of Pharmacy, Deraya University, Minia, Egypt; 3 Department of Pharmaceutics, Faculty of Pharmacy, Minia University, Minia, Egypt; 4 Department of Microbiology and Immunology, Faculty of Pharmacy, Badr University, Assuit, Egypt; 5 Department of Pharmaceutical Chemistry, Faculty of Pharmacy, Deraya University, Minia, Egypt; 6 Department of Pharmaceutics, Faculty of Pharmacy, Alexandria University, Alexandria, Egypt; 7 Department of Pharmaceutics, College of Pharmacy, King Khalid University, Abha, Saudi Arabia; Suez Canal University, EGYPT

## Abstract

Proteus mirabilis is a major uropathogen implicated in catheter-associated urinary tract infections and infection-induced urolithiasis, often exhibiting multidrug resistance. Improving the solubility and bioactivity of existing drugs represents a promising pharmaceutical strategy to overcome these challenges. In this study, inclusion complexes of metronidazole with α-cyclodextrin (α-CD), β-cyclodextrin (β-CD), and hydroxypropyl-β-cyclodextrin (HP-β-CD) were prepared using physical mixing and kneading methods. Complexes were characterized using differential scanning calorimetry (DSC) and Fourier-transform infrared spectroscopy (FTIR). In vitro release behaviour, minimum inhibitory concentration (MIC), and effects at sub-MIC levels on motility, urease-associated phenotype, and biofilm formation were evaluated. β-CD and HP-β-CD systems demonstrated improved release profiles compared to metronidazole alone. While MIC values remained within the mg/mL range, HP-β-CD complexes showed reduced MIC relative to aqueous metronidazole, achieving a four-fold reduction in MIC. At sub-MIC concentrations, β-CD and HP-β-CD formulations were associated with significant suppression of motility, reduced urease-associated phenotypes, and inhibition of biofilm formation. Molecular docking suggested spatial compatibility between metronidazole and cyclodextrins, though mechanistic conclusions remain predictive. These findings indicate that cyclodextrin-based complexation enhances physicochemical performance of metronidazole and may support adjunctive anti-virulence modulation in P. mirabilis. Further mechanistic and in-vivo studies are required to validate translational relevance.

## 1. Introduction

The rapid emergence of antibiotic resistance, attributed to the widespread use of antibiotics, especially the last-resort bactericidal antibiotics, is considered one of the major challenges facing human beings. There is a need to develop new antimicrobials or repurpose other non-antimicrobial drugs, searching for compounds that can enhance the efficacy of available antibiotics or those able to disrupt the virulence mechanisms of pathogens as a complementary strategy to weaken the pathogen and decrease its ability to induce infection. Recently, many researchers evaluated several compounds, such as mucolytics, analgesics, and natural compounds, for their antimicrobial and anti-virulence activity. As a complementary strategy, anti-virulence approaches have emerged as an alternative paradigm, rather than targeting bacterial viability. Anti-virulence agents aim to attenuate pathogens’ traits such as motility, toxin production, quorum sensing, biofilm formation, and enzyme secretion. By disarming pathogens without necessarily inhibiting growth, this strategy is proposed to impose lower evolutionary pressure and potentially preserve microbiome balance.

Metronidazole is a well-established nitroimidazole antimicrobial with potent activity against anaerobic microorganisms. Although it exhibits limited activity against aerobic pathogens, recent studies have suggested that metronidazole may modulate virulence traits in certain bacteria at sub-minimum inhibitory concentrations (sub-MICs). These findings raise the possibility of repurposing metronidazole as an adjunct anti-virulence agent rather than a primary antibacterial drug in infections caused by urease-producing organisms [1].

Khayyat, et al. [2] reported that Metronidazole can function as an anti-virulence and anti-quorum-sensing agent against *Proteus mirabilis* strains. In addition, it can decrease the MICs of some antibiotics against *Proteus mirabilis* strains. They reported that Metronidazole at sub-MIC can affect hemolysin production and biofilm formation. So, they suggested that it can be used in combination with antibiotics as an anti-virulence agent.

Urolithiasis, a common urinary tract condition, accounts for 1–13% of urinary stone cases, with various types of stones including calcium oxalate, uric acid, cystine, calcium phosphate, apatite, and struvite. Urinary stone formation can become problematic, especially in patients with catheterization showing infections with Proteus species, as it promotes the development of crystalline biofilms [3–13]. The treatment of infectious urolithiasis is complex, often requiring prolonged antibiotic therapy to eradicate bacteria and prevent stone recurrence. Fluoroquinolones are commonly recommended, but the challenge lies in antibiotics’ limited ability to penetrate stones where bacteria may persist. So, searching for urease inhibitors that can be used in combination with antibiotics can help in the eradication of infective stones [7,9,11,14–22].

However, metronidazole is slightly soluble in water, and its physicochemical limitations may restrict diffusion, bioavailability, and interaction with bacterial targets. Enhancing solubility may improve drug exposure at sub-MIC levels, where anti-virulence modulation is typically investigated. Furthermore, being given in large systemic doses, 500–1000 mg twice daily, and topical preparation up to 2% w/w necessitates developing more soluble forms and innovative enhancement of metronidazole solubility and antibacterial activities. In addition, metronidazole has poor organoleptic properties. Patients experience a bitter and metallic aftertaste [23]. Therefore, a single formulation approach can solve these three problems by adopting inclusion complexes of metronidazole and cyclodextrins to increase the solubility and the absorption of drugs and increase their diffusion through bacterial membranes [24].

While cyclodextrins are primarily used for solubility enhancement, emerging evidence suggests that improved physicochemical properties may indirectly influence antimicrobial performance by increasing effective drug exposure. Nevertheless, it is important to distinguish improved antimicrobial potency from genuine anti-virulence modulation. Anti-virulence assessment requires evaluation at sub-MIC levels and careful interpretation of phenotypic endpoints independent of growth inhibition.

Alpha (α-), β-, and γ-cyclodextrins are naturally occurring cyclic oligosaccharides (6- to 8-sugar moieties) having hydrophilic exteriors and hydrophobic centers. A doughnut-like form can be used to depict cyclodextrins schematically; its exterior is hydrophilic and highly soluble in water solutions. It has a hydrophobic interior. Cyclodextrins have been shown in more recent studies to improve antibacterial properties and fight multidrug resistance [25]. Promising tactics against bacterial resistance may be provided by cyclodextrins. These include lowering cholesterol levels, enhancing the penetration of antibacterial medications through bacterial cell walls, and limiting cell-to-cell communication [26]. Additionally, by reducing the activity of P-glycoproteins in the gastrointestinal membranes, cyclodextrins have shown inhibitory effects on efflux proteins [27].

By focusing on phenotypic suppression of virulence-associated traits rather than solely on bacterial growth inhibition, this study seeks to explore the potential of formulation-based modulation of an established drug as a complementary strategy in the management of catheter-associated P. mirabilis infections by evaluating the anti-virulence properties of metronidazole: cyclodextrins (α-, β-, and HP β-CDs) inclusion complexes against isolates of P. mirabilis that are extremely resistant.

## 2. Materials and Methods

Metronidazole was a courtesy free sample from Pharco Pharmaceuticals Corporation, Alexandria, Egypt. Alpha (α)-cyclodextrin, β-cyclodextrin and hydroxypropyl β-cyclodextrin were bought from Sigma-Aldrich, Saint Louis, MO, USA. All other chemicals and solvents were of an analytical grade and used as received.

### 2.1. Bacterial Strain

A Proteus mirabilis specimen was isolated from a patient suffering from urinary tract infection; it was Gram-stained and biochemically identified. Luria-Bertani (LB) broth and agar, Tryptone soya broth (TSB), and Mueller-Hinton (MH) broth and agar were purchased from Oxoid (Hampshire, UK). The isolated colonies were characterized biochemically. The isolate was Gram-negative rods, producing yellowish lactose non-fermenting colonies on MacConkey’s agar, positive indole and black-coloured butts of TSI tubes, and showing swarming on nutrient agar.

The microbial strain was obtained from the clinical laboratories of Assiut University Hospital (collected for routine lab diagnosis) and wasn’t directly obtained from patients (secondary use). Therefore, there was no direct or indirect contact with any patient, and thus no informed consent or ethical approval was required. The study protocol was conducted in accordance with the Declaration of Helsinki and prior approval (8/2024) by the Ethical Review Board of the Faculty of pharmacy, Deraya University, Egypt.

### 2.2. Molecular Docking Studies

Molecular docking studies were conducted using Molecular Operating Environment (MOE) 2014, 09 software (Chemical Computing Group, Montreal, QC, Canada) to predict the stability and possible orientation of metronidazole inside the cavity and/or rim of three cyclodextrins: α-CD, β-CD, and hydroxyl propyl (HP) β-CD. PDB file code 5E6Z was used to download the 3D structures of both α-CD and β-CD from the Protein Data Bank (PDB) at https://www.rcsb.org. Using the MOE software’s builder interface, the 3D structure of HPβ-CD was constructed by replacing the hydroxyl group with isopropyl radicals. After adding hydrogens, the QuickPrep tool in the MOE software was used to reduce the energy of CD structures to an RMSD (root mean square deviation) gradient of 0.01 kcal/mol. MOE Builder was also utilised to construct the 3D structures of the compounds. Using an induced-fit docking methodology that employed the Triangle Matcher method and the dG scoring system for pose ranking, compounds were docked into the inclusion cavity of the different CDs. The poses with the lowest binding free energy value and the maximum stability were chosen and reported after a visual evaluation of the resulting docking poses.

Further, molecular docking was performed to investigate the potential interaction of metronidazole with the active site of urease, a nickel-dependent metalloenzyme crucial for the pathogenicity of Proteus mirabilis and urolithiasis. The Klebsiella aerogenes urease C319A mutant complexed with acetohydroxamic acid (AHA) was used as the docking template (PDB ID: 1FWE), which features a well-characterized binuclear Ni^2+^ center essential for urease catalysis. The urease structure was prepared by eliminating water molecules and substituting hydrogen atoms. The active sites were defined to encompass the two Ni^2+^ ions (Ni574 and Ni575) and key residues, including His219, His272, and His134. For the docking of metronidazole, its 3D structure was built using the MOE builder and optimised using the same energy minimization protocols applied to the cyclodextrins. The docking of metronidazole was conducted using a rigid receptor-flexible ligand model. The docking grid was centered on the active site, and the induced-fit docking protocol was applied to account for the flexibility of the urease enzyme. The poses were ranked by their binding affinity (dG) and analyzed based on their interaction networks with the Ni^2+^ ions and the surrounding residues. The best poses were selected based on the lowest binding energy and stability, and these were used for further analysis of the ligand-receptor interactions, including metal coordination, hydrogen bonding, and proximity to catalytic residues.

### 2.3. Preparation of metronidazole: cyclodextrins, physical and kneaded mixtures

The equivalent of molar weights in milligrams (mg) of metronidazole and cyclodextrins (α-, β-, hydroxy propyl β-CDs) were weighed and mixed in a porcelain dish and a spatula for 5 minutes (min) to obtain a physical mixture. The obtained physical mixtures were passed through a metal sieve with an aperture size of 250 µm. For kneaded mixtures, the above procedures were repeated, and the final mixtures were kneaded with 1–2 mL of a hydromethanolic solution (50% v/v) till dough is obtained. The formed mass was pulverized and allowed to dry at ambient conditions and then passed through a metal sieve with an aperture size of 250 µm.

### 2.4. Differential scanning calorimetry (DSC)

Accurately weighed samples of 2–3 mg of metronidazole, metronidazole: CDs physical and kneaded mixtures were placed in aluminum pans. The temperature was raised from 30°C to 350°C at 10°C/min using a DSC calorimeter (PerkinElmer DSC 4000, the Netherlands) and Pyris Manger Properties software.

### 2.5. Fourier transform infrared spectroscopy (FTIR)

FTIR spectrophotometry (Agilent Cary 360 FTIR, Agilent Technologies, Malaysia) and Micro Lab FTIR software were used to stack the spectra of metronidazole, metronidazole: CDs physical and kneaded mixtures.

### 2.6. In vitro diffusion study

In vitro diffusion profiles of metronidazole and the prepared Met:CDs complexes were studied using the modified Franz diffusion model, a receptor compartment containing 12 mL of phosphate buffer pH 7.4 placed in a thermostatically shaking water bath adjusted at 37°C ± 1°C and 100 strokes per minute. A sample equivalent to 10 mg of metronidazole was dispersed in 2 ml of buffer and placed on the donor compartment, separated from the receptor compartment with a pre-soaked cellulose membrane (MW cut-off 12,000–14,000 Dalton). A sample of 1 mL was withdrawn at a predetermined time interval and measured at 310 nm for metronidazole using the Spectrogenics spectrophotometer, Thermo Scientific, USA.

### 2.7. Minimum inhibitory concentration (MIC)

The minimum inhibitory concentration of metronidazole dissolved in water and dimethyl sulfoxide (DMSO), each alone, and metronidazole-cyclodextrin (α-, β- and hp-CD) physical and kneaded mixture inclusion complexes were determined against the tested strain using the micro broth dilution method according to Clinical Laboratory and Standards Institute Guidelines (CLSI, 2023). Different concentrations ranging from 625 µg/mL to 2 × 104 µg/mL were used for the tested formulations. Briefly, an overnight culture of P. mirabilis was used and standardized to have turbidity equivalent to 0.5 McFarland with Muller-Hinton broth. Equal aliquots of serially diluted concentrations of the tested formulations and bacterial suspensions were added to wells of a 96-microtiter plate and incubated for 24h. The MIC was determined to be the lowest concentration that inhibits visible growth.

### 2.8. Assay of P. mirabilis motilities

Impact on the tested isolate’s swarming (SW), swimming (SM), and twitching (TM) motility: The amount of agar in the Luria-Bertani (LB) medium was suitable for the kind of motility being evaluated.

Swarming motility was evaluated using 0.7% agar media by applying 10 μL of the tested isolate cultured on the surface of agar. Swimming motility was evaluated using 0.3% of the agar media and the tested isolate was placed by puncturing into the medium. Twitching motility was assessed using 1.0% agar and 10 μL of the tested isolate was inoculated by micropipette under the agar surface. Sub-MIC concentrations of the samples studied were added to the prepared media before the application of the organism to assess their ability to inhibit different forms of mobility. After cultivation for 24 h at 37°C, the diameters of the inhibition zones were recorded.

### 2.9. Urease activity assay

This assay was investigated to assess the inhibitory effects of metronidazole on the synthesis of the urease enzyme [20]. In the center of Christensen’s urea agar plates supplemented with the tested agents (metronidazole dissolved in water and DMSO separately, metronidazole: β-CD, metronidazole: α-CD, and metronidazole: HP β-CD) at ½ MIC, 5 µL of P. mirabilis overnight cultures were impeded. The plates were then incubated at 37^o^ C for twenty-four hours. The urease activity was indicated by the pH indicator changing from yellow to pink. Drugs were not added to control plates, which were made in the same way. The pink zones that were created were measured in millimeters. The experiment was conducted in triplicate, and the urease inhibition percentages were computed.

### 2.10. Effects of metronidazole and metronidazole:cyclodextrin-dispersed mixtures on biofilm formation by Proteus mirabilis

The effects of the prepared samples on the adherence of the tested strain and their ability to form a biofilm were assessed. The biofilms of P. mirabilis were generated in 96-well polystyrene plates (Tarson, India) employing the methods reported by Wei, et al. [28]. The overnight-grown culture was used to prepare microbial suspensions, and the suspension’s turbidity was adjusted to 0.7 OD610 (1 × 10⁹ CFU/ml). In tryptone soy broth (TSB) (Difco Laboratories) with 0.5% glucose added, sub-MIC concentrations of the investigated combinations and metronidazole at a concentration of each alone were made. One hundred µL of TSB broth supplemented with 0.5% glucose was placed in each well. Sub-MIC concentrations of tested drugs were added, followed by the addition of 10 μL of the microbial suspension. Plates were incubated for 18 h at 37 °C, washed using PBS; the biofilms were fixed using methanol for 15–30 min and then stained with crystal violet. The biofilm formation was assessed by adding ethanol 95% to the crystal violet-stained wells. The absorbance was measured at 593 nm using a microplate reader (Multiskan spectrum; Finland)


$$\begin{array}{c} \text{Percentage of inhibition }(\%)\;=\text{ 100 }-\;\left(\frac{{OD}_{s}}{{OD}_{C}}\right)x\;\text{1}\text{0}\text{0} \end{array}$$

Where OD_S_ and OD_c_ are the absorbance measured at 595 nm for the sample and control, respectively.

### 2.11. Inclusivity in global research

“Additional information regarding the ethical, cultural, and scientific considerations specific to inclusivity in global research is included in the Supporting Information."

### 2.12. Statistical Analysis

All experiments were performed in triplicate, and results are presented as mean ± standard deviation (SD). Statistical significance between groups was determined using one-way analysis of variance (ANOVA) followed by Tukey’s post-hoc test for multiple comparisons. A p-value < 0.05 was considered statistically significant. GraphPad Prism (version 8.0.1, GraphPad Software, USA) was used for data analysis and figure generation.

## 3. Results

### 3.1. In silico prediction of metronidazole-cyclodextrins complexes

**Fig 1:**
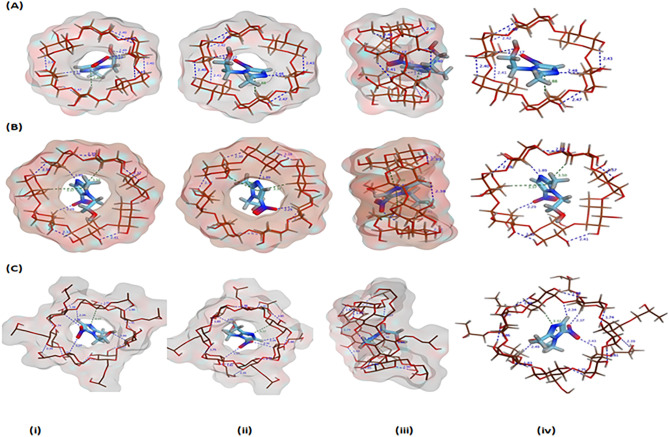
Three-dimensional poses of metronidazole docked into the inclusion cavities of (A) α-CD, (B) β-CD, and (C) HP β-CD showing (i) top view, (ii) bottom view, (iii) side view, and (iv) top view with marked interactions showing bond lengths.

Molecular docking was performed to explore the potential spatial compatibility of metronidazole within the cavities of α-cyclodextrin (α-CD), β-cyclodextrin (β-CD), and hydroxypropyl-β-cyclodextrin (HP-β-CD). Docking scores ranged between −4.68 and −5.06 kcal/mol (Table 1), values consistent with weak-to-moderate non-covalent host–guest interactions typically observed in cyclodextrin inclusion systems. Metronidazole was predicted to orient within the cyclodextrin cavity through hydrogen bonding involving hydroxyl and nitro functional groups, as well as hydrophobic contacts between the imidazole ring and the internal hydrophobic surface of the cyclodextrins. HP-β-CD demonstrated slightly lower binding energy values compared with native β-CD, potentially reflecting enhanced accommodation due to hydroxypropyl substitution and increased cavity flexibility (Fig 1). These results, representing docking simulations, provide predictive insights under simplified computational conditions that suggest possible compatibility between metronidazole and cyclodextrins

### 3.2. FTIR

**Fig 2:**
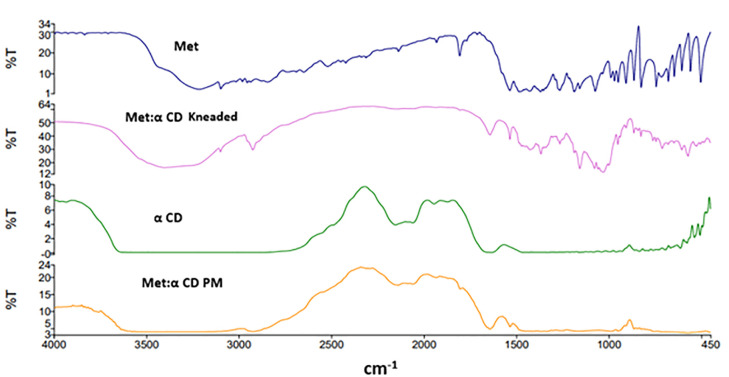
FTIR spectra of metronidazole (Met), α-CD, Met: α-CD physical mixture (PM) and Met: α-CD kneaded dispersion.

**Fig 3:**
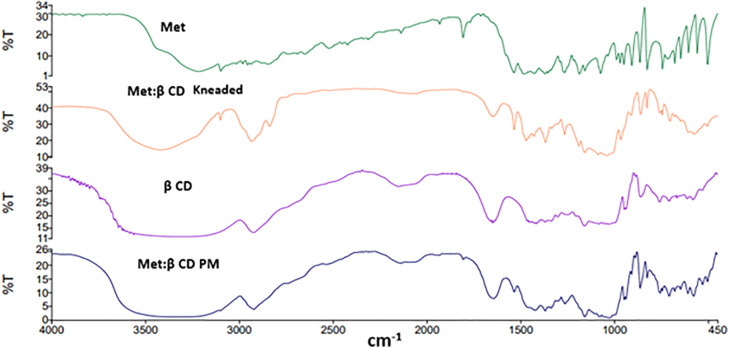
FTIR spectra of metronidazole (Met), β-CD, Met: β-CD physical mixture (PM) and Met: β-CD kneaded dispersion.

**Fig 4:**
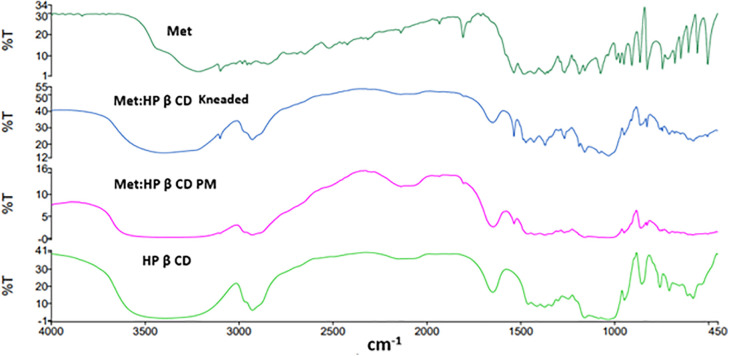
FTIR spectra of metronidazole (Met), HP β-CD, Met: HP β-CD physical mixture (PM) and Met: HP β-CD kneaded dispersion.

Figs 2-4 show FTIR spectra of metronidazole (Met), the used cyclodextrins, their physical mixtures (PM) and coprecipitated dispersions (Coppt).

The FTIR spectra of Met showed characteristic vibrational peak for C-H stretching at 2991 cm^-1^. IR peaks at 1600 and 1523 cm^-1^ were assigned to imidazole group C = C and C = N stretching, respectively. A broad peak between 3186−3300 cm^-1^ was assigned to OH (alcohol) stretching (Fig 2 and Fig 3). The N = O asymmetric stretching was assigned to a peak at 1478 cm^-1^, the methylene bending vibration peak was assigned to a peak at 1461−1451 cm⁻¹, and the carbon-carbon stretching peak was assigned to peak at 1423−1423 cm⁻¹.. The absorption peaks at 1389 and 1359 cm^-1^ were assigned to methyl bending and N = O asymmetric stretching, respectively. Further, absorption peaks at 1271−1097, 1071 and 721 cm⁻¹ were assigned to C-O stretching, C-N stretching, and =C-H bending, respectively.

The FTIR spectra of cyclodextrins demonstrated broad peaks at 3200–3550 cm⁻¹, indicating stretching of the hydroxyl groups and the stretching vibration of –CH- from 1610 to 1716 cm⁻¹. Intense peaks of 2854 cm⁻¹ were recorded for CDs due to -C-H asymmetric/symmetric stretching vibration. FTIR spectra of PM always serve as a control and represent the superimposition of the Met and cyclodextrin spectra alone (Fig 4). The characteristic vibrational peaks of Met (e.g., -N stretching, -CH₂- bending vibration and =C-H bending) were weakened/disappeared could be attributed to hydrophobic moieties in cyclodextrin cavities

### 3.3. DSC

**Fig 5:**
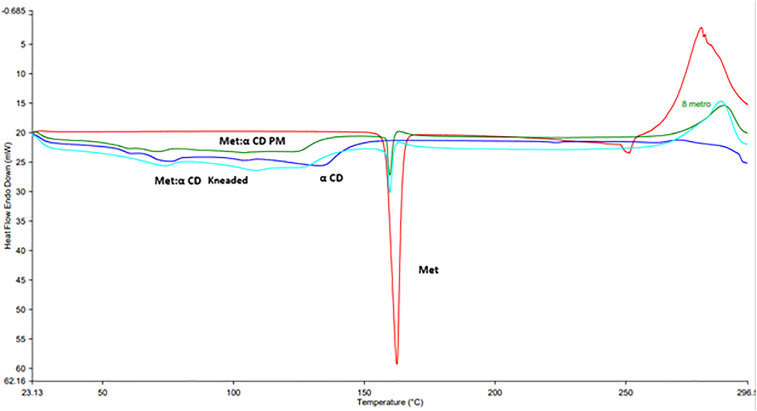
DSC thermograms of metronidazole (Met), α-CD, Met: α-CD physical mixture (PM) and Met: α-CD kneaded (Coppt) dispersion.

**Fig 6:**
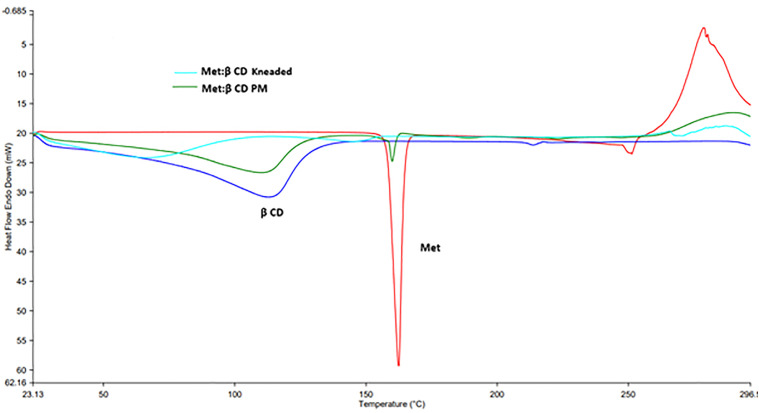
DSC thermograms of metronidazole (Met), β-CD, Met: β-CD physical mixture (PM) and Met: β-CD kneaded dispersion.

**Fig 7:**
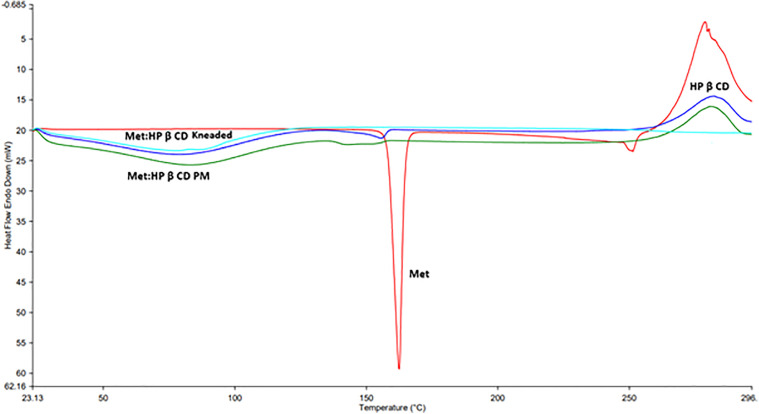
DSC thermograms of metronidazole (Met), HP β-CD, Met: HP β-CD physical mixture (PM) and Met: HP β-CD kneaded dispersion.

Figs 5–7 displayed the thermograms of Met, cyclodextrins, their PM mixtures and Coppt dispersions. A strong endothermic peak appeared at 160°C, which was attributed to metronidazole melting indicating the highly crystalline nature of metronidazole powder. Both Met: α-CD PM and Coppt showed small peaks of a markedly lower intensity, indicating loosening of the crystalline lattice/microcrystallites formed (Fig 5). Similarly, Met: β-CD PM showed a weak residual melting peak of metronidazole. Complete disappearance of the melting peak of Met was observed with Met:β-CD PM stronger interactions and complete inclusion complexation (Fig 6). On the contrary, both Met: HP β-CD PM and Met: HP β-CD Coppt displayed the complete disappearance of the crystalline Met peaks. These results correlated well with in silico prediction/molecular docking studies. Strongest interactions and inclusion complexes were formed with Met-HP β-CD mixtures (Fig 7).

### 3.4. In vitro diffusion study

**Fig 8:**
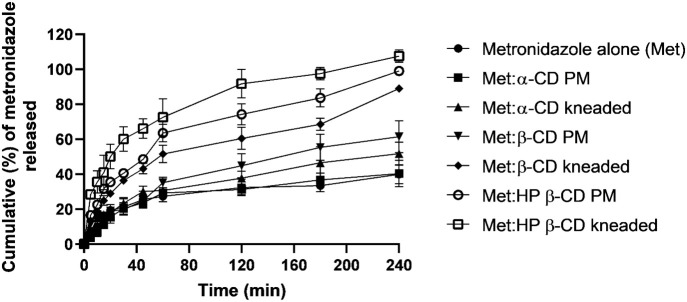
In vitro diffusion profiles of metronidazole and metronidazole from different Met:CD dispersed mixtures prepared by physical mixing (PM) and kneading methods.

The release profiles (Fig 8) revealed that metronidazole alone exhibited poor solubility, reaching only ~50% dissolution after 4 hours. Inclusion complexes with β-CD and HP β-CD significantly enhanced dissolution, achieving complete release (>95%) within 2 hours (p < 0.001 vs. metronidazole alone). In contrast, α-CD complexes showed no significant improvement (p > 0.05). These results correlated well with those obtained from the in silico investigation, FTIR, and DSC data, indicating a poor/unfitted inclusion between metronidazole and the small cavity-bearing α-CD. This was evident from the slow and incomplete diffusion of metronidazole (approximately 50% dissolved within 4 h). On the contrary, both Met:β-CD and Met:HP β-CD mixtures (irrespective of the method of preparation) showed proportional marked increases of drug diffusion or release rates based on energy scores of complexes between the drug and cyclodextrins’ cavities (Table 1). For example, faster and complete (up to 100%) diffusion was recorded for both Met: β-CD and Met: HP β-CD mixtures.

**Table 1:** Energy scores and the number of possible contacts recorded for metronidazole docked into the inclusion pocket of α-cyclodextrin, β-cyclodextrin, and hydroxypropyl-β-cyclodextrin.

Host	Energy scores (Kcal/mol)	No. of possible interactions	
**H bond**	**Hydrophobic interactions**
α-cyclodextrin	−4.6843	3	1
β-cyclodextrin	−5.0075	2	2
Hydroxypropyl β-cyclodextrin	−5.0594	4	1

### 3.5. Determination of MIC

The MICs for metronidazole alone, metronidazole: CDs physical and kneaded mixtures inclusion complexes were assessed against the isolated P. mirabilis isolate by the broth microdilution method. The MIC values against P. mirabilis are summarized in Table 2. Metronidazole dissolved in water showed MIC > 2 × 10⁴ µg/ml, while solubilization in DMSO improved activity to 1 × 10⁴ µg/ml. Complexation with α- and β-CDs resulted in MIC values similar to DMSO-solubilised metronidazole (p > 0.05). Notably, HP β-CD complexes reduced the MIC to 5 × 103 µg/ml, representing a two-fold improvement over DMSO-metronidazole (p < 0.01) and a four-fold improvement over aqueous metronidazole (p < 0.001). These results correlated well with silico molecular docking, thermal analysis, and FTIR. Irrespective of the method of preparation, metronidazole: HP β-CD prepared by either physical mixing or the kneading method showed the lowest MIC values (Table 2). Similar results were recorded elsewhere and highlighted the importance of antibiotic solubility in the diluent/solvent used [29]. Further, increased antibiotic uptake by complexation with CDs could explain the lower MIC (5 × 103 µg/ml) than using DMSO (1 × 104 µg/ml). Similar results were recorded with cyclodextrin complexation with various antibiotics such as β-lactams, quinolones, tetracyclines, aminoglycosides, nitroimidazoles, and oxazolidinones [30].

**Table 2:** The MIC values estimated for metronidazole and metronidazole: cyclodextrins PM and kneaded mixtures using *P. mirabilis* isolates are as follows:.

Studied agents	MIC × 10^3^ (µg/ml)
Metronidazole (Met) dissolved in water	20
Met dissolved in DMSO	10
Met: α-CD (PM & kneaded)	10
Met: β-CD (PM & Kneaded)	10
Met: HP β-CD (PM & Kneaded)	5

In the current study, we investigated the ability of metronidazole—a clinically established antimicrobial agent—to suppress the virulence factors of P. mirabilis, particularly urease activity, motility, and biofilm formation. Notably, metronidazole exhibited poor solubility in water and limited efficacy when used alone. However, its formulation with cyclodextrins (CDs), particularly β-cyclodextrin (β-CD) and hydroxypropyl-β-cyclodextrin (HP β-CD), significantly improved its solubility and biological activity.

### 3.6. Effect on bacterial motilities

The reduction in bacterial motility caused by metronidazole suggests that the drug has anti-virulence properties. Bacterial motilities are essential for adhesion and biofilm development. The bacterial motilities recorded were swarming, twitching, and swimming.

#### 3.6.1. Effect on swarming motility.

**Fig 9:**
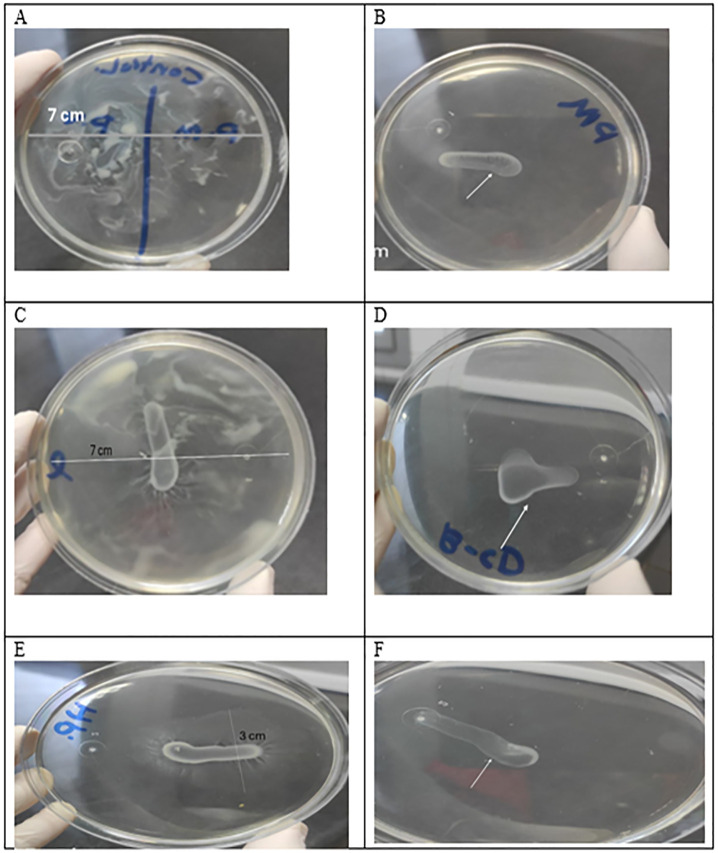
Effects of the samples investigated on the swarming of Proteus mirabilis. A: control, B: in the presence of metronidazole in DMSO (no swarming), C: in the presence of metronidazole: α-CD (showing residual swarming activities), D: in the presence of metronidazole: β-CD (no swarming), E: in the presence of metronidazole: HP β-CD (SD) (swarming area decreased to 3 cm). F: in the presence of Metronidazole: HP β-CD (PM) (no swarming).

**Fig 10:**
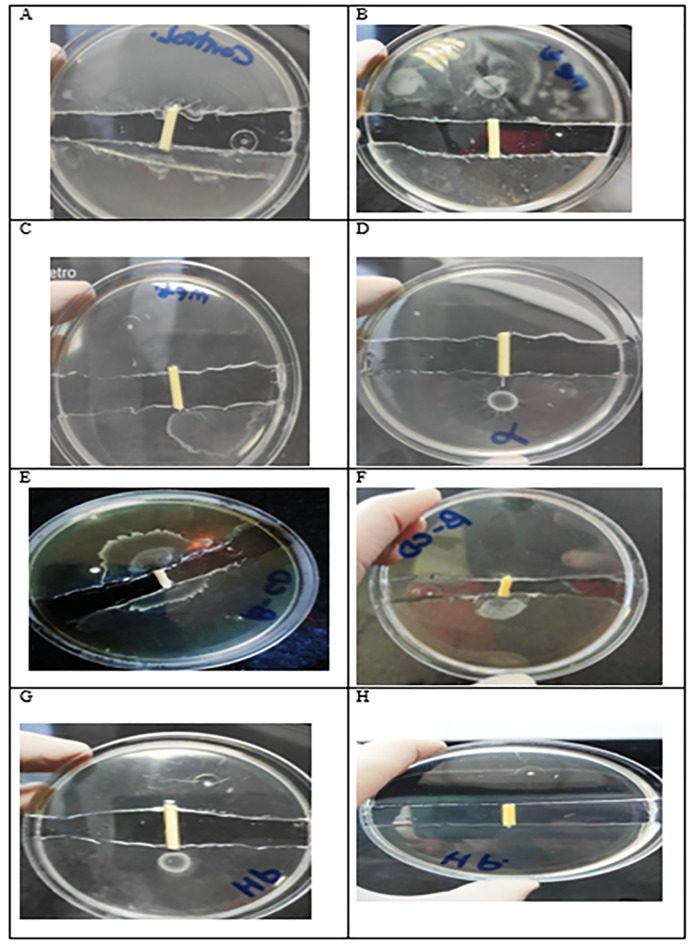
Effects of the investigated samples on the swarming of Proteus mirabilis over catheter pieces. A: control, B: in the presence of metronidazole dissolved in water (swarming); C: in the presence of metronidazole dissolved in DMSO (no swarming). D: in the presence of metronidazole: α-CD (swarming), D: in the presence of metronidazole: β-CD (kneaded) (swarming shown in a decreased distance compared to control); E: Swarming was observed in the presence of metronidazole-β-CD (SD). F: in the presence of metronidazole: β-CD (PM) (no swarming). G: In the presence of metronidazole: HP β-CD (PM) (no swarming). H: in the presence of metronidazole: HP-β-CD (PM) (no swarming).

By evaluating the changes in the ability of Proteus mirabilis to move in the presence of cyclodextrins (α-, β- and HP β-CD) and metronidazole, Table 3, Fig 9 & Fig 10 showed photographs and scoring of swarming activities, respectively, using control (untreated), metronidazole, and metronidazole: CDs treated agar Petri dishes. Metronidazole dissolved in water had a minimal effect on swarming. It can inhibit swarming on the agar surface but failed to inhibit swarming over the catheter piece. In contrast, DMSO-solubilized metronidazole inhibited swarming on both the agar surface and over the catheter piece. HP-β-CD, β-CD, and α-CD (both PM and kneaded) complexes dissolved in water significantly suppressed swarming on both surfaces compared to metronidazole dissolved in water (p < 0.001). These results agreed with those obtained from DSC and FTIR studies.

**Table 3:** Effect of the tested compounds on the swarming on the agar surface and over the catheter pieces.

Studied Compounds		Swarming motility		P-value
		On agar surface	Over catheter pieces	
Control (without drug)		70 mm	+	
Metronidazole	Water	No swarming	+	*P < 0.05* ^*^
	DMSO	No swarming	–	*P < 0.001* ^**^
Met: α-CD (PM & kneaded)		No swarming	–	
Met: β-CD	kneaded	No swarming	–	
	PM	No swarming	–	
Met: HP β-CD	Kneaded	No swarming	–	
	PM	No swarming	–	

#### 3.6.2. Effect on twitching motility.

**Fig 11:**
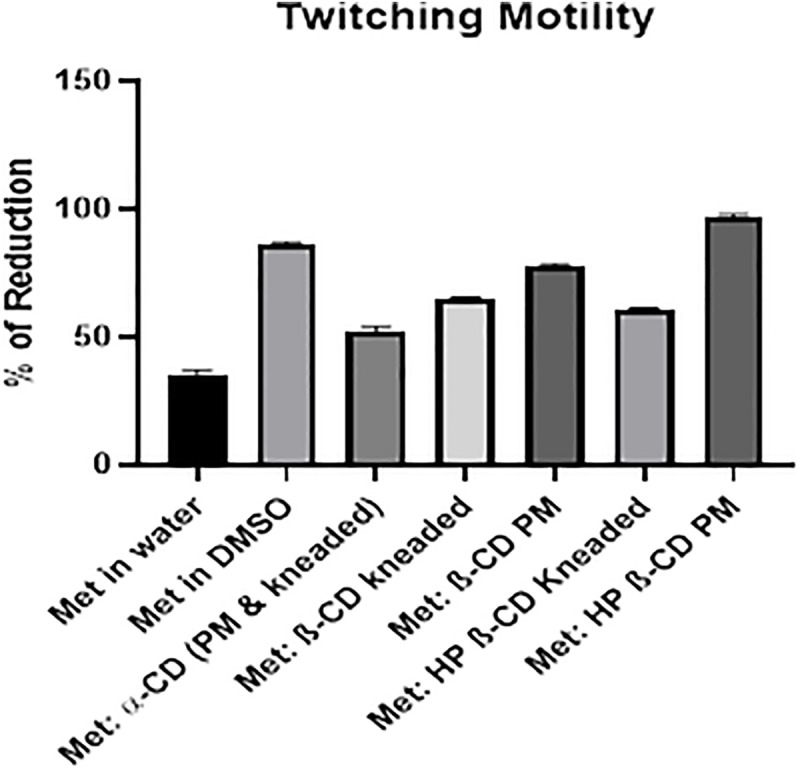
Effect of the tested compounds on the twitching motility of the tested isolate.

**Fig 12:**
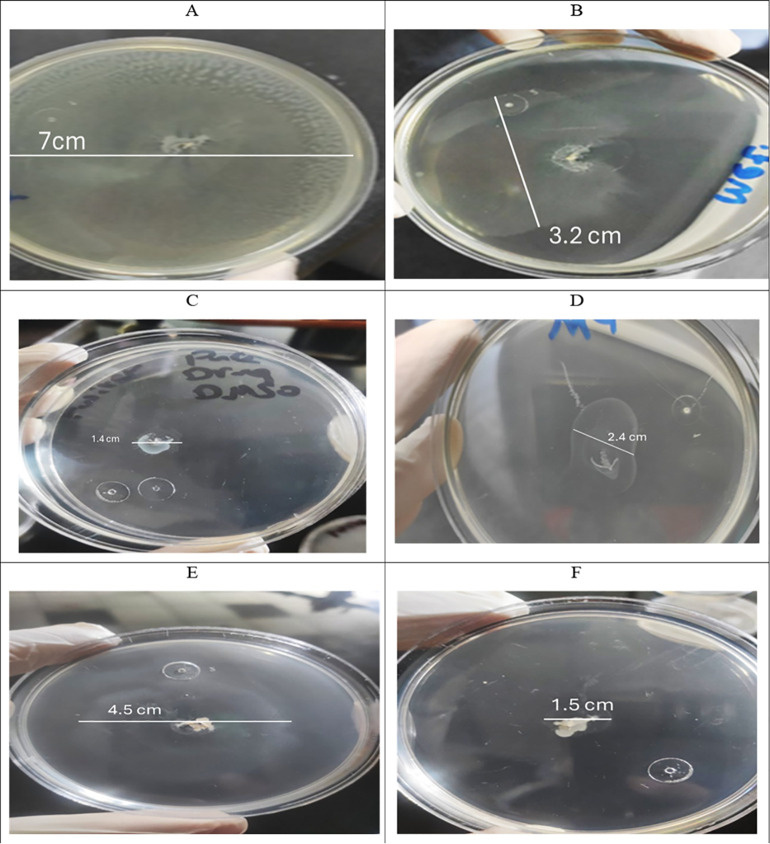
Effect of the investigated samples on the twitching of Proteus mirabilis. A: control, B: in the presence of metronidazole dissolved in water, C: in the presence of metronidazole dissolved in DMSO, D: in the presence of metronidazole: α-CD, E: in the presence of metronidazole: β-CD (kneaded), F: in the presence of metronidazole: β-CD (PM), G: in the presence of metronidazole: HP-β-CD (kneaded), H: in the presence of metronidazole: HP-β-CD (PM).

Fig 11. showed significant inhibition of the twitching motility by HP-β-CD inclusion complexes, followed by metronidazole dissolved in DMSO & Met:-β-CD (PM) (97.1, 86 & 77.8% reduction in comparison to control, respectively) (P < 0.001). In contrast, Metronidazole dissolved in water, and the α-CD:metronidazole inclusion complex showed the lowest inhibitory effect in comparison to the control. Fig 12 G–12H.

#### 3.6.3. Effect on swimming motility.

**Fig 13:**
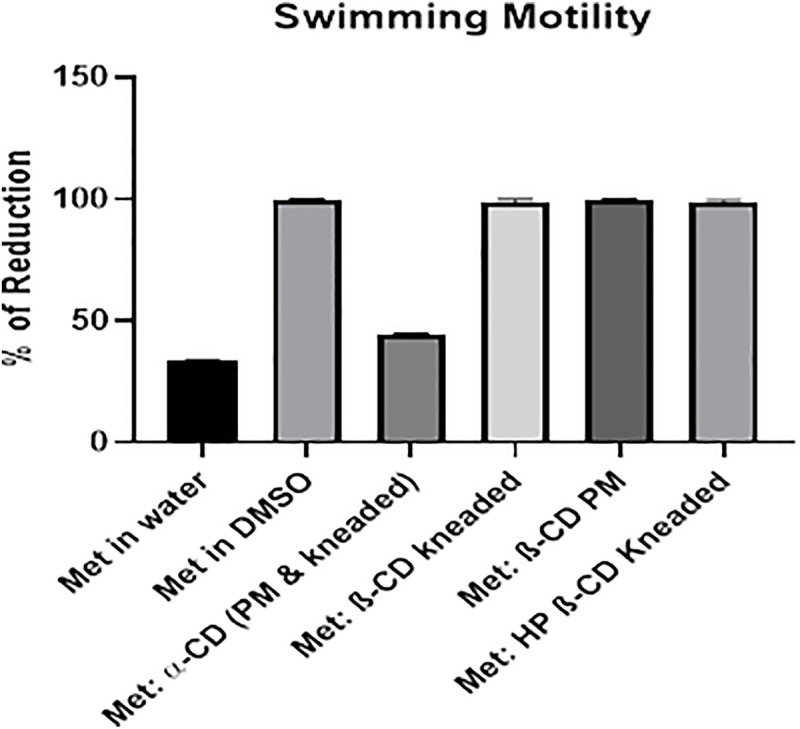
Effect of the tested compounds on the swimming motility of the tested isolate.

**Fig 14:**
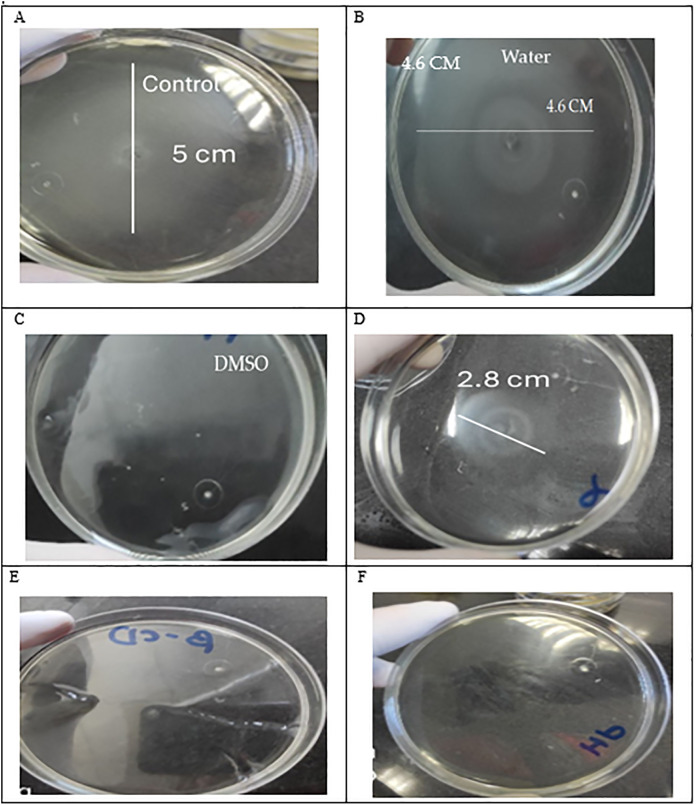
Effect of the tested agents on the swimming motility of Proteus mirabilis. A: control, B: in the presence of metronidazole dissolved in water, C: in the presence of metronidazole dissolved in DMSO, D: in the presence of metronidazole: α-CD, E: in the presence of metronidazole: β-CD (PM), F: in the presence of metronidazole: HP-β-CD (PM).

For swimming motility, Metronidazole in DMSO, metronidazole:HP β-CD PM, and metronidazole:β-CD PM completely inhibited swimming motility of the tested isolate in comparison to the control (P < 0.001). Met: α-CD inclusion complex showed lower inhibitory activity close to that shown by Metronidazole dissolved in water, as shown in Figs 13 & Fig 14.

### 3.7. Effect on the urease enzyme

**Fig 15:**
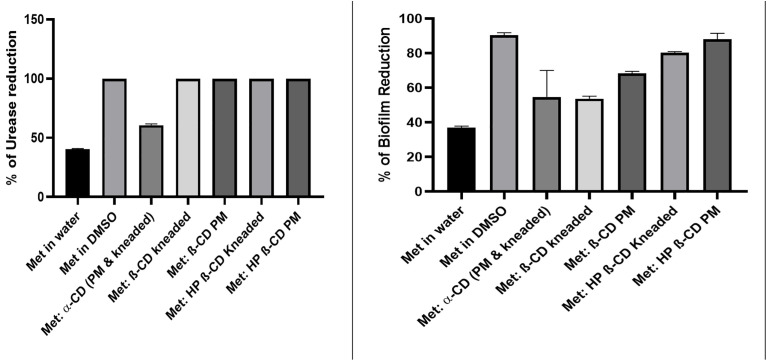
Effects of the samples investigated on urease enzyme production and biofilm formation.

**Fig 16:**
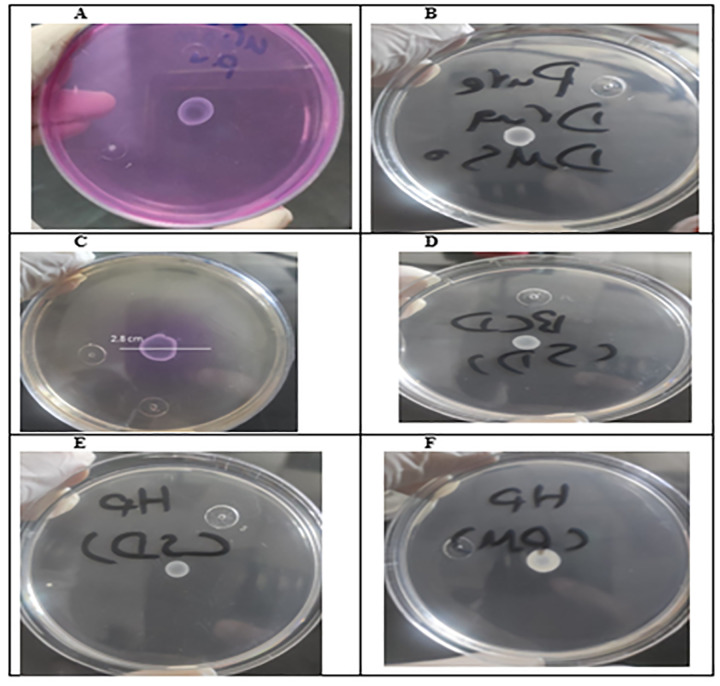
Effects of the samples investigated on urease enzyme production by Proteus mirabilis. A: control, B: in the presence of metronidazole dissolved in DMSO, C: in the presence of metronidazole: α-CD, D: in the presence of metronidazole: β-CD (kneaded), E: in the presence of metronidazole: HP- βCD (kneaded), F: in the presence of metronidazole: HP-βCD (PM).

The impact of metronidazole and its cyclodextrin formulations on urease-associated phenotype was evaluated using Christensen’s urea agar. Untreated control plates demonstrated the expected color change from yellow to pink, indicating active urea hydrolysis. Metronidazole dissolved in water & α-CD tested systems produced a limited reduction in colour production due to incomplete solubility of the drug, as explained above in sections 3.4 and 3.5.1 (Figs 15 & Fig 16). In contrast, metronidazole dissolved in DMSO, as well as β-CD and HP-β-CD formulations (both physical and kneaded systems), showed a marked decrease in the intensity and extent of the colour change (urease-associated phenotype rather than enzymatic inhibition) (P < 0.0001). The HP-β-CD-containing formulation demonstrated the most pronounced suppression. The mechanism underlying this reduction may involve altered metabolic activity, reduced virulence expression, or enhanced drug exposure following solubility improvement. Further studies employing purified urease enzyme assays and kinetic analysis would be required to determine whether direct catalytic inhibition occurs or a urease-associated phenotype [31].

### 3.8. In silico prediction of metronidazole-cyclodextrins’ anti-urease and anti-virulence effects

**Fig 17:**
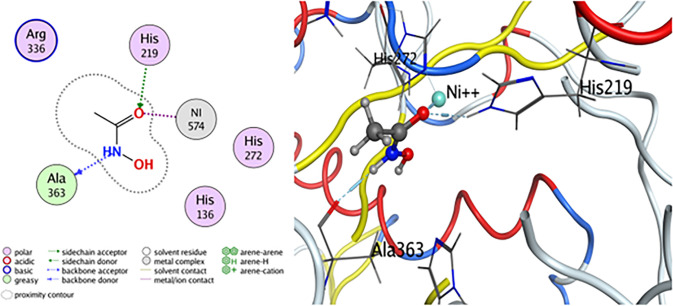
3D and 2D interaction diagrams showing acetohydroxamic acid (AHA) docked into the urease active site (PDB ID: 1FWE). 3D rendering of AHA coordinating Ni²⁺ ion (Ni574) with hydrogen bonding to His219 and Ala363. 2D interaction plot showing key contacts, including metal chelation and hydrogen bonding.

To explore whether metronidazole could potentially interact with urease, molecular docking was performed using the crystal structure of Klebsiella aerogenes urease (PDB ID: 1FWE). The crystal structure of Klebsiella aerogenes urease C319A mutant in complex with acetohydroxamic acid (AHA) (PDB ID: 1FWE) was retrieved from the Protein Data Bank and used as the docking template. This structure is considered a gold standard for modelling bacterial urease inhibition due to its well-characterized nickel (Ni^2+^) active site, which is conserved across bacterial species, including *Proteus mirabilis* [32]. The protein was processed by removing water molecules, adding polar hydrogens, and assigning appropriate charges. The ligand structures (metronidazole and AHA) were energy-minimized and converted to appropriate formats. The docking grid was centered around the active site, encompassing the two Ni^2+^ ions and key residues including His219, His272, and His134. A flexible ligand–rigid receptor protocol was employed, and binding affinities were scored based on calculated free energy and visual inspection of interaction networks. As a benchmark for validating the docking procedure, acetohydroxamic acid (AHA), a well-known urease inhibitor, was redocked into the active site. The docking reproduced the experimental pose with high fidelity, yielding a binding energy of −8.41 kcal/mol and a root mean square deviation (RMSD) of 1.10 Å. The ligand exhibited chelation with the Ni^2+^ ion, and it formed key hydrogen bonds with His219 and Ala36. These interactions suggest the essential features of effective urease inhibition and support the robustness of the docking protocol (Fig 17).

**Fig 18:**
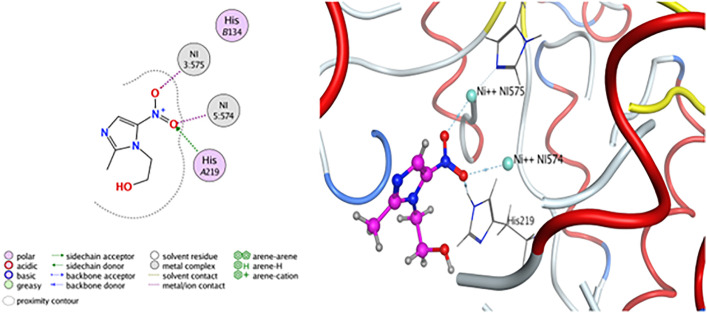
3D and 2D docking interactions of metronidazole with the urease active site (PDB ID: 1FWE). £D binding pose metronidazole may occupy a region proximal to the active site and form hydrogen bonding interactions with surrounding amino acid residues.

The molecule displayed a binding energy of −7.95 kcal/mol and an RMSD of 1.43 Å, indicating a stable binding pose. A stabilising hydrogen bond acceptor was observed between the nitro oxygen and His219, a crucial active-site residue responsible for catalytic function. The imidazole ring and hydroxyethyl side chain were nestled within the hydrophobic pocket, further stabilizing the ligand (Fig 18). As a result, docking suggested that metronidazole may occupy a region proximal to the active site and form hydrogen bonding interactions with surrounding amino acid residues.

Unlike AHA, which functions as a classical transition-state analogue, metronidazole acts as a non-classical inhibitor with a different pharmacophoric motif. However, it still fulfils the crucial requirement of Ni^2+^ engagement and interaction with catalytic residues, suggesting that it may inhibit urease through competitive mechanisms.

## 4. Discussion

Our study investigated whether cyclodextrin-based inclusion complexation could improve the physicochemical performance of metronidazole and influence virulence-associated phenotypes of uropathogenic *Proteus mirabilis*, rather than positioning metronidazole as a potent antibacterial agent against this organism. So, we discussed the interpreted results as a formulation-enhanced adjunctive framework, where solubility improvement may facilitate modulation of virulence traits at sub-inhibitory concentrations.

Molecular docking offers a tool for the prediction of potential characteristics of complexes formed with cyclodextrins in the literature. In the current work, molecular docking anticipated the formation of stable complexes between the small-sized metronidazole and the examined cyclodextrins, as suggested by the negative value of the energy scores listed in Table 1. In accordance with the experimental results, the order of the stability complexes formed was that with α-cyclodextrin < β-cyclodextrin < hydroxypropyl-β-cyclodextrin.

FTIR spectral analysis is one of the characterization techniques to support in silico/molecular docking studies and inclusion complexation by interpreting the shift of vibrational peaks of key functional groups of FTIR spectra. These results correlated well with that from the in silico prediction/molecular docking studies. Metronidazole may occupy a region proximal to the active site and form hydrogen bonding interactions with surrounding amino acid residues. Similarly, in vitro dissolution of metronidazole from the prepared cyclodextrin complexes demonstrated potential enhancement of the dissolution rate and extent from Met:β-CD and Met:HP-β-CD mixtures.

The minimum inhibitory concentrations (MICs) observed in this study remained within the mg/mL range, even after cyclodextrin complexation. Although HP-β-CD complexes demonstrated a reduction in MIC compared with aqueous metronidazole, these absolute values remain substantially higher than those typically reported for clinically potent antibacterial agents. Therefore, the translational relevance of metronidazole as a standalone antibacterial therapy against P. mirabilis appears limited under the conditions investigated.

Instead, the more relevant observations relate to phenotypic modulation detected at sub-MIC levels. Anti-virulence strategies aim to attenuate pathogenic traits without necessarily inhibiting bacterial growth. In this context, the observed reduction in motility, suppression of urease-associated phenotypes, and inhibition of biofilm formation represents virulence-associated endpoints distinct from bactericidal activity. Furthermore, determination of MIC values followed by evaluation at sub-MIC concentrations is a commonly employed approach in studies investigating the effects of drug combinations or formulation strategies on bacterial virulence traits. Several researchers have used this approach to assess how antimicrobial agents or drug–carrier complexes influence pathogenic behaviours such as motility, enzyme production, and biofilm formation in various bacterial species [30,33,34].Swarming, swimming, and twitching motilities are critical for surface colonization, catheter migration, and biofilm establishment. Sub-MIC exposure to metronidazole formulations—particularly those containing HP-β-CD—was associated with reduced motility phenotypes. This effect may result from improved drug exposure due to enhanced solubility, facilitating intracellular penetration, or interference with regulatory pathways or a reflection of quorum sensing modulation, metabolic interference, stress-response induction, or indirect growth-related alterations. Similarly, the potential effect of HP-β-CD-containing systems on suppressing urease-associated phenotypes may reflect metabolic modulation or altered bacterial physiology rather than direct active-site inhibition. Biofilm formation is a complex, multi-step process involving adhesion, motility, quorum signalling, and extracellular matrix production. Our study showed that HP-β-CD-containing systems demonstrated the strongest inhibition of biofilm biomass at sub-MIC concentrations. Enhanced solubility likely increases effective drug availability during early attachment stages. Additionally, cyclodextrins themselves have been reported to interact with membrane components and potentially alter lipid microenvironments at higher concentrations. Improved drug exposure and altered physicochemical interactions collectively contribute to observed biofilm suppression [24,25,35,36].

## Results

According to these results, HP β-CD and the β-CD inclusion complex (PM) with metronidazole have the best activity against the tested strain and inhibitory activity against the tested virulence factors, followed by α-CD [37]. Similar results regarding the effect of HP β-CD and the β-CD inclusion complex effect on the anti-virulence activity of some antibiotics were reported. Choi, et al. [38] showed that ciprofloxacin with a β-CD inclusion complex derivative can enhance the ability of ciprofloxacin to inhibit growth and MRSA biofilm. Also, HP β-CD-functionalized cellulose gauzes containing vancomycin and the QSI HAM are capable of affecting S. aureus and P. aeruginosa biofilms [39]. Oprea, et al. [40] reported that the release of the biologically active form of the fourth-generation cephalosporin Cefepime from the ZnO matrix may help reduce the risk of biofilm-related infections by preventing bacterial adhesion, subsequent colonization, and biofilm formation on a variety of surfaces.

Cyclodextrin inclusion systems are commonly interpreted within the framework of Higuchi and Connors phase solubility theory, where host–guest complex formation is classified into AL-, AP-, or AN-type profiles depending on the shape of the solubility curve. Although formal phase solubility experiments were not performed in the present study, the enhanced release behavior observed for β-CD and HP-β-CD systems is most consistent with an AL-type system, characterized by a linear increase in drug solubility as a function of cyclodextrin concentration.

AL-type profiles typically suggest the formation of a 1:1 host–guest inclusion complex, where solubilization increases proportionally without evidence of higher-order aggregation. The improved release observed with HP-β-CD compared to α-CD aligns with known cavity size compatibility, as the β-CD cavity (~6–6.5 Å) is more suitable for accommodating small heterocyclic compounds such as metronidazole than the smaller α-CD cavity (~4.7–5.3 Å) [41–43]

While HP-β-CD demonstrated superior performance in the present study, alternative β-cyclodextrin derivatives such as sulfobutylether-β-cyclodextrin (SBE-β-CD) and randomly methylated β-cyclodextrin (RM-β-CD) have also been reported to enhance the solubility of poorly water-soluble drugs. SBE-β-CD, in particular, possesses an anionic character that may further improve aqueous compatibility and reduce aggregation tendencies. RM-β-CD offers increased lipophilicity and may enhance membrane interaction in certain systems [44,45]. However, these derivatives may differ in regulatory status, toxicity profile, and cost, which influence formulation selection. HP-β-CD remains one of the most widely accepted derivatives due to favourable safety data and established pharmaceutical use.

To gain further insight into the mechanism of action, we employed molecular docking simulations using MOE 2014.09 software. Initially, docking studies suggested the formation of stable inclusion complexes between metronidazole and α-, β-, and HP β-CDs. The ranking of complex stability, based on binding free energy, followed the order: α-CD < β-CD < HP-β-CD, which supports our in vitro activity results. These results validate the role of CD-based complexation in enhancing the biological performance of metronidazole. Docking of metronidazole into the same site revealed promising binding characteristics. These interactions suggest that metronidazole may function as a non-classical urease inhibitor, disrupting enzyme function through alternative binding geometries compared to traditional inhibitors. This novel mechanism of action supports the urease-phenotype suppression effects observed experimentally and expands the therapeutic scope of metronidazole beyond its known antimicrobial properties. This dual experimental and computational investigation highlights the potential of metronidazole-CD complexes as promising candidates for adjunctive therapy against catheter-associated UTIs and infectious urolithiasis caused by urease-producing pathogens.

### Limitations of this study

Several limitations of the present study should be acknowledged. First, phase solubility analysis was not performed to formally classify the cyclodextrin inclusion system (e.g., AL-type profile) or determine stability constants, which would provide additional thermodynamic insight into the complexation process. Second, the in vitro release experiment employed a Franz diffusion cell with a dialysis membrane, which reflects combined drug release and diffusion rather than intrinsic dissolution alone. Third, advanced structural confirmation techniques such as PXRD or ROESY-NMR were not included. Fourth, urease activity was assessed using a phenotypic Christensen’s urea agar assay; therefore, the results indicate suppression of the urease-associated phenotype rather than direct enzymatic inhibition, which requires purified enzyme kinetics studies. Fifth, the study was conducted entirely under in vitro conditions, and no in vivo model was used to evaluate pharmacokinetics, urinary dilution effects, catheter flow dynamics, or physiological drug exposure. Finally, although complexation reduced MIC values, the absolute MICs remained relatively high compared with conventional antibacterial agents, which limits the interpretation of metronidazole as a standalone antibacterial therapy against *P. mirabilis*. Accordingly, the findings should be interpreted as a proof-of-concept demonstration of formulation-driven enhancement and phenotypic virulence modulation, rather than as definitive therapeutic validation.

### Future research perspective

Future investigations should focus on comprehensive phase solubility analysis, structural confirmation of inclusion complexes using advanced techniques such as PXRD or NMR, and enzymatic assays to validate potential urease inhibition mechanisms. In addition, evaluation in physiologically relevant infection models—including catheter-associated urinary tract infection models and pharmacokinetic studies—will be essential to determine the clinical relevance of the observed anti-virulence modulation.

## 5. Conclusion

This study demonstrates that complexation of metronidazole with β-cyclodextrin and hydroxypropyl-β-cyclodextrin improves its release behaviour and modulates selected virulence-associated phenotypes of uropathogenic Proteus mirabilis in vitro. Although the absolute antibacterial potency remained limited, enhanced solubility was associated with reduced motility, suppression of urease-associated phenotypes, and inhibition of biofilm formation at sub-MIC concentrations. The findings suggest that formulation-driven optimization of existing drugs may support adjunctive anti-virulence strategies rather than serve as standalone antibacterial therapies. However, mechanistic conclusions regarding direct enzyme inhibition or molecular interactions remain speculative and require biochemical validation. Additionally, the absence of in vivo validation limits immediate translational applicability.

## Supporting information

S1 Data Inclusivity in global research questionnaire. (PDF)

S2 Data GraphPad Prism file showing dissolution profile data of metronidazole and Metronidazole: cyclodextrin different mixtures prepared by physical mixing and kneading mixtures. (PZFX)

S3 Data GraphPad Prism file for the effect of the tested compounds on motility, biofilm and urease production. (PZF)

S4 Data Three-dimensional interaction diagram showing interaction of metronidazole: α-CD with urease active site. (JPG)

S5 Data Three-dimensional interaction diagram showing interaction of metronidazole: β-CD with urease active site. (JPG)

S6 Data Three-dimensional interaction diagram showing interaction of metronidazole: HP β-CD with urease active site. (JPG)
