# Supplementary figures and images for: Cyclodextrin inclusion complexes enhance the solubility and anti-virulence activity of metronidazole against uropathogenic Proteus mirabilis
Source: PLoS One. 2026 Jul 27;21(7):e0353058. doi: 10.1371/journal.pone.0353058 (PMC13405122; doi:10.1371/journal.pone.0353058)

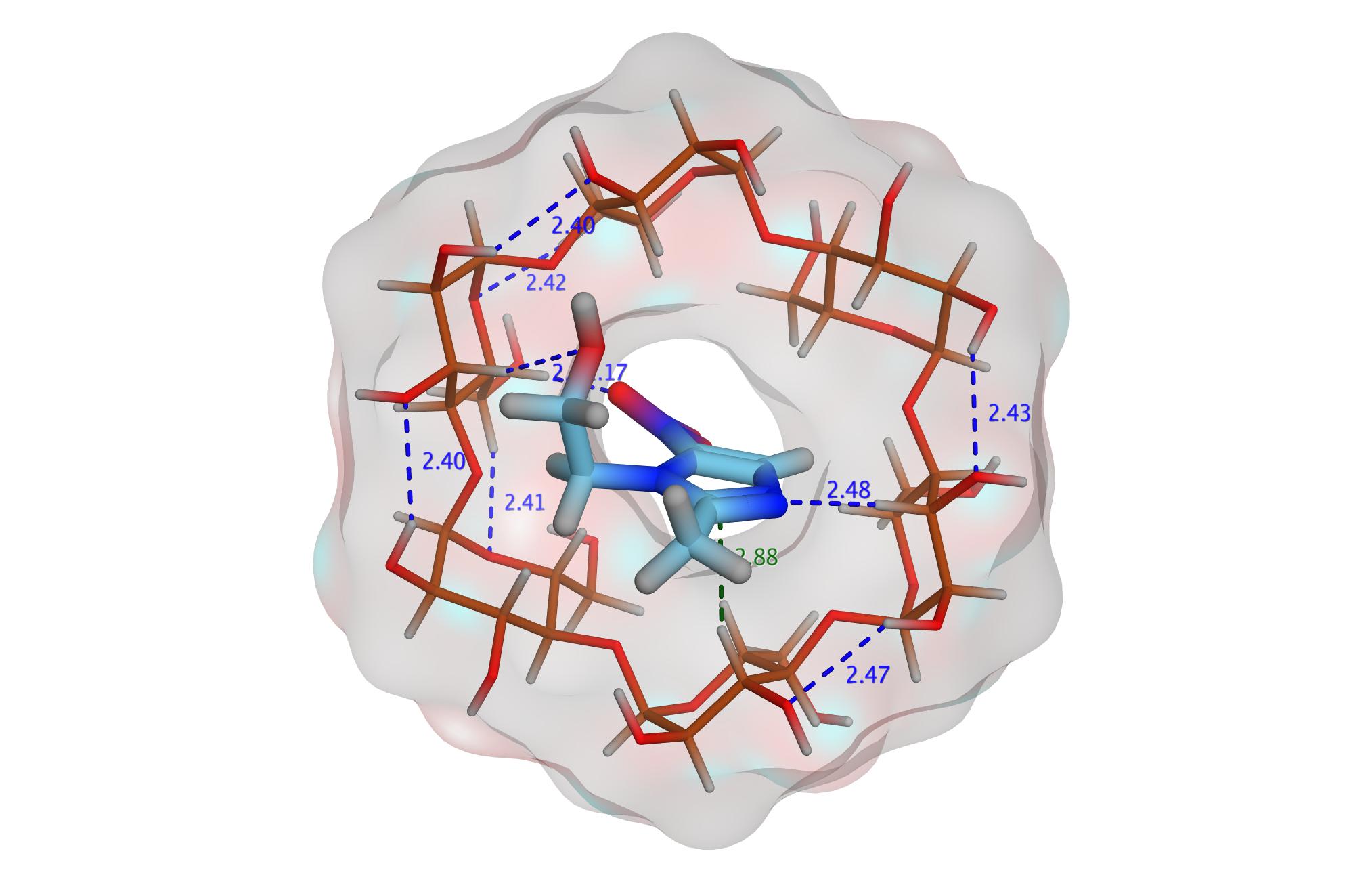

Supplement: S4 Data — (JPG) [file pone.0353058.s004.jpg]

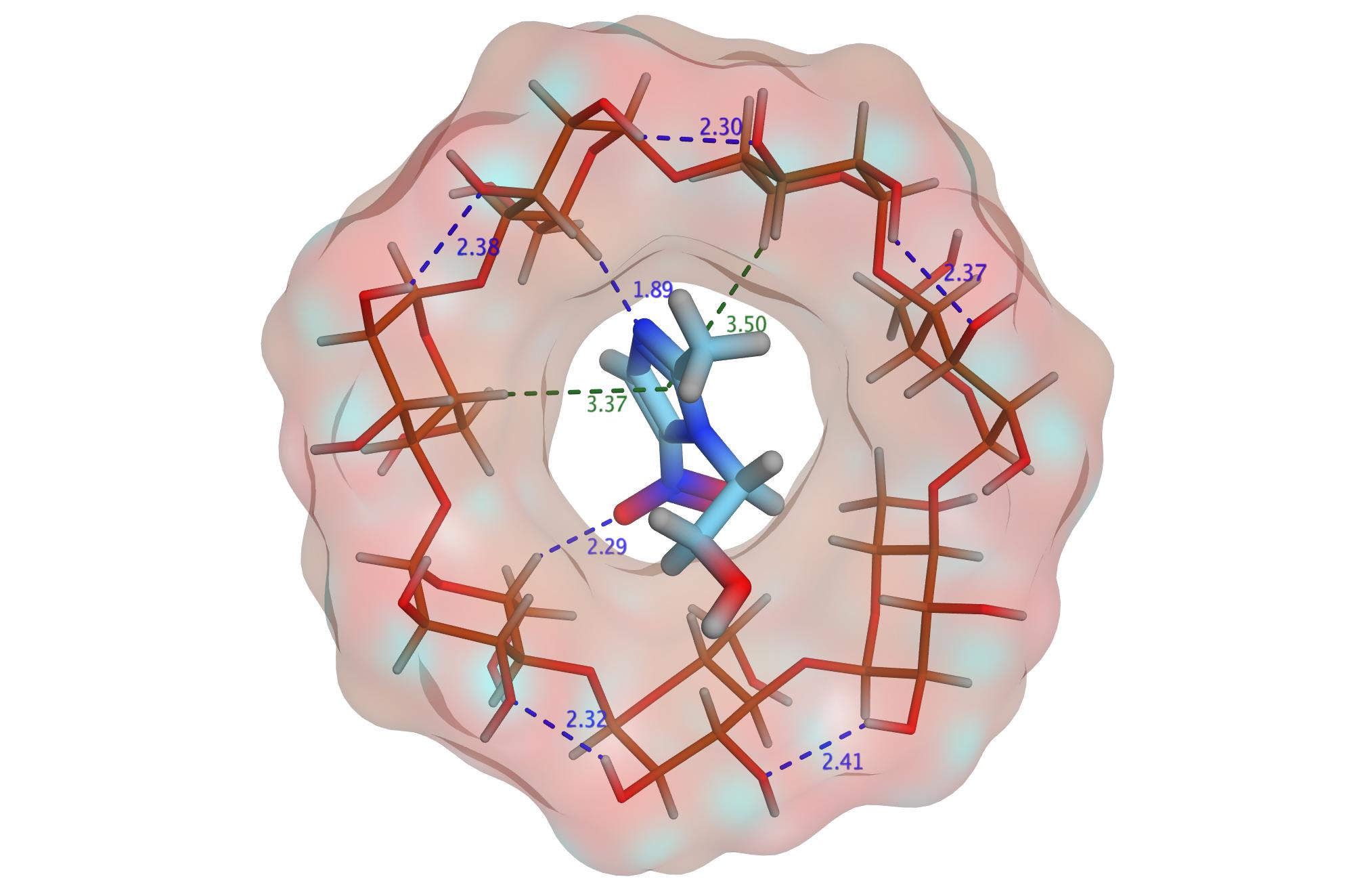

Supplement: S5 Data — (JPG) [file pone.0353058.s005.jpg]

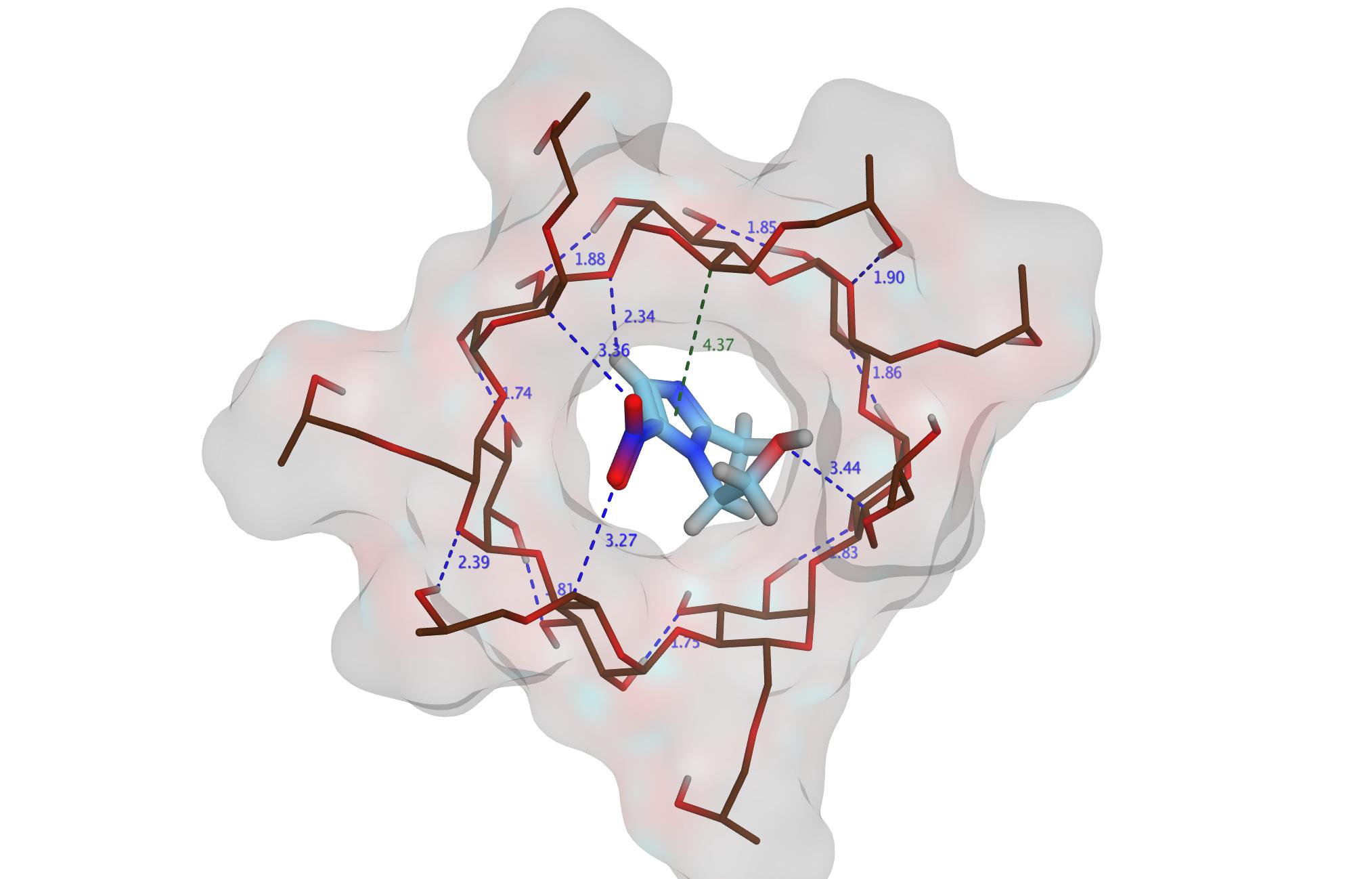

Supplement: S6 Data — (JPG) [file pone.0353058.s006.jpg]
