# Supplementary material for: Cyclodextrin inclusion complexes enhance the solubility and anti-virulence activity of metronidazole against uropathogenic Proteus mirabilis
Source: PLoS One. 2026 Jul 27;21(7):e0353058. doi: 10.1371/journal.pone.0353058 (PMC13405122; doi:10.1371/journal.pone.0353058)
Supplement: Attachment — Submitted filename: Authors reply.docx [file pone.0353058.s008.docx]

**Dear Editor,**

We sincerely thank you and the reviewers for the thorough evaluation of our manuscript entitled:

**“Cyclodextrin Inclusion Complexes Enhance the Solubility and Anti-Virulence Activity of Metronidazole Against Uropathogenic *Proteus mirabilis*.”**

We have carefully revised the manuscript in response to all reviewers' and editors' comments. Major revisions include:

- Clear conceptual distinction between antibacterial and anti-virulence activity throughout the manuscript.
- Substantial reduction of mechanistic overstatement regarding molecular docking and urease interaction.
- Reframing urease findings as suppression of urease-associated phenotype rather than confirmed enzymatic inhibition.
- Clarification that the in vitro Franz diffusion system reflects release/diffusion behavior rather than intrinsic dissolution alone.
- Addition of a dedicated “Limitations of the Study” subsection.
- Contextual discussion of absolute MIC values and translational relevance.
- Condensation and tightening of the molecular docking sections.
- Professional English language editing throughout.

**Ethics Statement Clarification**

The study involved the secondary use of a clinical bacterial isolate obtained from laboratory diagnostics. No patient identifiers were accessed. The Ethics Committee of the Faculty of Pharmacy, Deraya University (Approval No. 8/2024) reviewed the protocol and waived the requirement for informed consent, as the study involved anonymized microbial samples without direct or indirect patient contact.

The ethics statement now appears only in the Methods section, in accordance with journal requirements.

**Funding Statement Amendment**

The Funding Statement has been amended to read:

“This work was funded by the Deanship of Research and Graduate Studies at King Khalid University through Small Group Research under grant number RGP 1/4/46. The funders had no role in study design, data collection and analysis, decision to publish, or preparation of the manuscript.”

It is a contractual obligation to acknowledge the Funding information

**Data Availability Confirmation**

All raw data underlying the findings, including replicate values used for statistical analysis and figure generation, are provided in the Supporting Information files. This includes raw values for MIC assays, motility measurements, urease-associated phenotype assays, biofilm quantification, and release/diffusion studies.

**Authorship**

The authorship list has been updated to include all listed authors as requested.

**Figures**

All figures are original and were generated by the authors. No copyrighted third-party material has been used. For example: these plates are from those in the manuscript before cropping edges

|  |  |
| --- | --- |

We believe the revised manuscript now addresses all scientific, conceptual, and editorial concerns raised during peer review. We sincerely appreciate the opportunity to improve our work and look forward to your further consideration.

Thank you for your time and careful evaluation.

Sincerely,

**Prof. Rehab Mahmoud Abd El-Baky**
Corresponding Author
Faculty of Pharmacy
Minia University & Deraya University
Email: rehab.mahmoud@mu.edu.eg

Point to point reply

| Reviewer comments | Author reply |
| --- | --- |
| Reviewer #1: |  |
| The manuscript is well written and organized. Figures are well demonstrated with good reflection.  Statistical results are mentioned properly.  Pharmacokinetic parameters are not given. You can add PK study in this paper | We thank the reviewer for the encouraging comments and valuable suggestions.  The present study was designed as an **in vitro pharmaceutical and anti-virulence investigation**, focusing on cyclodextrin complexation, physicochemical characterization, release behavior, and phenotypic modulation of virulence factors. Therefore, **in vivo pharmacokinetic (PK) experiments were not included within the scope of the current work**.  To address this point, we have now added a paragraph in the **Discussion section (Limitations of the Study)** acknowledging the absence of pharmacokinetic evaluation and highlighting it as an important direction for future research. Specifically, we state that future studies should evaluate pharmacokinetics and urinary drug concentrations in relevant **in vivo infection models**. |
|  |  |
| Reviewer #2: | We sincerely thank the reviewer for the detailed and insightful critique. The manuscript has been substantially revised to address all conceptual, methodological, and interpretational concerns. |
| The study "Cyclodextrin Inclusion Complexes Enhance the Solubility and Anti-Virulence Activity of Metronidazole Against Uropathogenic Proteus mirabilisis" is methodologically sound and internally consistent, and the experimental dataset is substantial. However, over‑interpretation, conceptual ambiguity, and insufficient critical depth currently limit its suitability for publication without major revision. Following are the major concerns and recommendations for authors. **Reviewer 2: Major Scientific Concerns**:   1. Anti virulence vs. Antibacterial Activity   The manuscript repeatedly alternates between antibacterial efficacy and anti virulence activity without clearly delineating the two concepts.  - MIC reduction is used as evidence of improved “anti virulence” activity, yet MIC is a growth inhibition metric, not a virulence specific endpoint.  - True anti virulence strategies are typically defined by effects at sub MIC levels without affecting growth. While sub MIC experiments are mentioned later (motility, urease, biofilm), the manuscript does not consistently frame or interpret results within this paradigm. | We agree with the reviewer that the conceptual distinction between antibacterial activity and anti-virulence modulation required clearer presentation.  The manuscript has been revised as follows:   - The **Introduction section has been rewritten** to clearly define anti-virulence strategies. - A clear distinction between **MIC (growth inhibition)** and **sub-MIC anti-virulence endpoints** has been introduced. - MIC reduction is now interpreted as **improved antimicrobial exposure resulting from cyclodextrin complexation**, rather than direct anti-virulence activity. - Motility, urease-associated phenotype, and biofilm assays are now explicitly framed as **sub-MIC anti-virulence evaluations**.   These changes improve conceptual clarity throughout the manuscript. |
| 2. Over‑Interpretation of Molecular Docking Results - Docking scores of −4 to −5 kcal/mol for CD inclusion are modest and fall within a range where enthalpy–entropy compensation and solvent effects dominate. Claiming “perfect fit” or “strongest interactions” is overstated. - The assertion that metronidazole’s nitro group chelates both Ni²⁺ ions in urease similarly to acetohydroxamic acid is highly speculative. Nitroimidazoles are not established urease chelators in vivo. - No experimental urease kinetics (IC₅₀, mode of inhibition) are provided to validate docking predictions. | We appreciate this important observation.  The molecular docking section has been carefully revised to avoid over-interpretation:   - Statements such as **“perfect fit” and “strongest interactions”** have been removed. - Docking scores (−4 to −5 kcal/mol) are now described as **indicative of possible compatibility between metronidazole and cyclodextrin cavities rather than strong binding interactions**. - Claims suggesting **Ni²⁺ chelation within the urease active site** have been removed. - A paragraph discussing **limitations of molecular docking**, including solvent effects and enthalpy–entropy compensation, has been added. - Docking findings are now described as **predictive and hypothesis-generating rather than mechanistically confirmatory**.   Additionally, duplicated docking descriptions were **condensed into a single subsection**. |
| 1. Misinterpretation of FTIR and DSC as Proof of Inclusion FTIR peak “weakening/disappearance” and DSC melting point suppression are repeatedly described as proof of complete inclusion complexation. - FTIR spectral overlap with cyclodextrins frequently masks drug peaks even in simple physical mixtures. - DSC melting peak disappearance can result from amorphization or dilution, not necessarily inclusion. Authors can support these results with complementary techniques (PXRD, phase solubility diagrams, Job’s plot, NMR ROESY). | We agree.  We have:   - Removed statements claiming “proof of complete inclusion”. - Replaced them with “consistent with possible inclusion complex formation”. - Added a paragraph acknowledging that PXRD, ROESY-NMR, or phase solubility studies would provide stronger evidence. - Added this as a limitation of the current work. |
| 2. Dissolution Methodology and Interpretation The “in vitro dissolution” experiment uses a Franz diffusion cell with a dialysis membrane, which measures diffusion + dissolution, not classical dissolution. This setup is more representative of permeation release than intrinsic solubility. - Interpretation as dissolution enhancement alone is therefore misleading. Rename this experiment to “in vitro release/diffusion study” and clarify its limitations. Consider acknowledging that CDs may alter membrane transport rather than dissolution alone. | Correct.  We have:   - Renamed the section to “In vitro release/diffusion study”. - Clarified the experimental setup. - Explained that results reflect release across a membrane rather than intrinsic solubility alone. - Added a limitations statement. |
| 3. MIC Values Remain Extremely High Even after formulation, MIC values remain in the mg/mL range (5–20 mg/mL), which is:  - Orders of magnitude higher than clinically relevant antibiotic MICs - Not discussed critically in terms of translational relevance Claiming “significant enhancement” without contextualizing these absolute values risks overstatement. Discuss clinical relevance candidly. Emphasize anti‑virulence potential at sub‑MIC levels rather than antimicrobial potency. | We agree with the reviewer and have revised the Discussion accordingly.  The manuscript now explicitly acknowledges that the observed MIC values remain within the **mg/mL range**, which is substantially higher than those typically reported for clinically potent antibiotics.  Therefore, the study is now clearly framed within an **anti-virulence modulation context**, emphasizing that the most relevant findings relate to **phenotypic modulation observed at sub-MIC concentrations**, including:   - motility reduction - suppression of urease-associated phenotype - inhibition of biofilm formation   These endpoints represent **virulence modulation rather than bactericidal activity**. |
| 4. Urease Assay Interpretation The Christensen’s urea agar assay measures phenotypic urease activity, not direct enzyme inhibition. - Reduced color change may reflect growth suppression, altered metabolism, or diffusion limitations, not enzyme inhibition. - Yet results are discussed as “urease enzyme inhibition.” Rephrase throughout to “suppression of urease activity/expression” rather than enzyme inhibition, unless biochemical assays are added. | We agree with the reviewer.  The manuscript has been revised as follows:   - The term **“urease inhibition”** has been replaced with **“suppression of urease-associated phenotype.”** - The text now clarifies that Christensen’s urea agar measures **phenotypic urease activity under growth conditions rather than direct enzymatic inhibition**. - Statements implying direct catalytic inhibition have been removed. |
| 5. Missing areas, author need to add out come of these 1. Phase solubility theory (AL/AN/AP types)– fundamental to CD inclusion science. 2. Comparative discussion of CD derivatives beyond HP‑β‑CD (e.g., SBE‑β‑CD, RM‑β‑CD). 3. Limitations of cyclodextrins (cholesterol extraction, membrane toxicity at high concentrations). 4. Recent anti‑virulence frameworks (QS inhibition vs metabolic interference). 5. In vivo relevance– no discussion of urinary dilution, urine composition, or catheter flow dynamics. 6. Results are restated rather than critically analyzed. - Contradictions are not explored (e.g., α‑CD showing activity despite poor inclusion). - No comparison is made with alternative solubility enhancers or non‑CD systems.  - Why HP‑β‑CD outperforms β‑CD beyond solubility (steric shielding, hydration shell). - Whether observed anti‑virulence effects stem from CDs themselves. What are the limitations of docking‑driven mechanistic ?. 7. Data Presentation and Interpretation - Molecular docking content is duplicated across sections. Condense docking description into a single cohesive subsection and tighten the Discussion. 8. Professional language editing is strongly advised. 9. Limitations - Conceptual novelty is incremental, not transformative. - No new formulation principle or mechanistic model is proposed.   Specific Recommendations for Improvement  1. Rewrite Introduction to clearly define anti virulence vs antibacterial activity.  2. Soften mechanistic claims from docking and FTIR/DSC.  3. Clarify dissolution vs diffusion methodology.  4. Reframe urease results as phenotypic suppression.  5. Add a limitations subsection in Discussion.  6. Tighten language and reduce redundancy.  7. Consider removing or shortening repetitive docking descriptions.  ________________________________________ | We thank the reviewer for this helpful suggestion.   - **Phase solubility theory** based on the Higuchi–Connors model (AL-type systems) extensibvely studied and the main foculs of this manuscript is to study the effect of cyclodextrins pn the antibacterial activities of metronidazole but not solubility. - **Cyclodextrin is regarded as safe excipients but the issue of cholesterol extraction if applied on mucous membrane such as eye or injected and the toxicity of these excipients is beyond the current investigation.** - **Comparison with alternative cyclodextrin derivatives**, including SBE-β-CD and RM-β-CD - **Potential limitations of cyclodextrins**, including membrane cholesterol extraction and concentration-dependent toxicity - Integration of **anti-virulence conceptual frameworks**, including phenotypic modulation and metabolic interference mechanisms   These additions strengthen the theoretical context of the study.  The manuscript has undergone **professional language editing** to improve clarity, grammar, and overall readability.  We agree that the study represents an **incremental but meaningful formulation-based investigation**.  To address this point, a **dedicated “Limitations of the Study” subsection** has been added in the Discussion, outlining:   - absence of phase solubility experiments - lack of enzyme kinetics validation - in vitro experimental design - need for future in vivo pharmacokinetic and infection model studies   We sincerely thank the reviewers for his recommendations and comments, which significantly improved the clarity, balance, and scientific rigor of our manuscript.  All recommendations were taken in consideration in response and modifying our manuscript |
