# Supplementary material for: Cyclodextrin inclusion complexes enhance the solubility and anti-virulence activity of metronidazole against uropathogenic Proteus mirabilis
Source: PLoS One. 2026 Jul 27;21(7):e0353058. doi: 10.1371/journal.pone.0353058 (PMC13405122; doi:10.1371/journal.pone.0353058)
Supplement: Peer review history

# Peer review: Decision Letter 0

**DOI:** 10.1371/journal.pone.0353058.r001

**Authors:** Reham Mokhtar ELTarabili

2 Mar 2026

PONE-D-25-60159

Cyclodextrin Inclusion Complexes Enhance the Solubility and Anti-Virulence Activity of Metronidazole Against Uropathogenic Proteus mirabilis

PLOS One

Dear Dr. Abd El-Baky,

Thank you for submitting your manuscript to PLOS ONE. After careful consideration, we feel that it has merit but does not fully meet PLOS ONE’s publication criteria as it currently stands. Therefore, we invite you to submit a revised version of the manuscript that addresses the points raised during the review process.

Please submit your revised manuscript by Apr 16 2026 11:59PM. If you will need more time than this to complete your revisions, please reply to this message or contact the journal office at plosone@plos.org. When you're ready to submit your revision, log on to https://www.editorialmanager.com/pone/ and select the 'Submissions Needing Revision' folder to locate your manuscript file.

Please include the following items when submitting your revised manuscript:

- A letter that responds to each point raised by the academic editor and reviewer(s). You should upload this letter as a separate file labeled 'Response to Reviewers'.
- A marked-up copy of your manuscript that highlights changes made to the original version. You should upload this as a separate file labeled 'Revised Manuscript with Track Changes'.
- An unmarked version of your revised paper without tracked changes. You should upload this as a separate file labeled 'Manuscript'.

If you would like to make changes to your financial disclosure, please include your updated statement in your cover letter. Guidelines for resubmitting your figure files are available below the reviewer comments at the end of this letter.

If applicable, we recommend that you deposit your laboratory protocols in protocols.io to enhance the reproducibility of your results. Protocols.io assigns your protocol its own identifier (DOI) so that it can be cited independently in the future. For instructions see: https://journals.plos.org/plosone/s/submission-guidelines#loc-laboratory-protocols. Additionally, PLOS ONE offers an option for publishing peer-reviewed Lab Protocol articles, which describe protocols hosted on protocols.io. Read more information on sharing protocols at https://plos.org/protocols?utm_medium=editorial-email&utm_source=authorletters&utm_campaign=protocols.

We look forward to receiving your revised manuscript.

Kind regards,

Reham Mokhtar ELTarabili

Academic Editor

PLOS One

Journal Requirements:

When submitting your revision, we need you to address these additional requirements.

1. Please ensure that your manuscript meets PLOS ONE's style requirements, including those for file naming. The PLOS ONE style templates can be found at

https://journals.plos.org/plosone/s/file?id=wjVg/PLOSOne_formatting_sample_main_body.pdf and https://journals.plos.org/plosone/s/file?id=ba62/PLOSOne_formatting_sample_title_authors_affiliations.pdf

2. Please include a complete copy of PLOS’ questionnaire on inclusivity in global research in your revised manuscript. Our policy for research in this area aims to improve transparency in the reporting of research performed outside of researchers’ own country or community. The policy applies to researchers who have travelled to a different country to conduct research, research with Indigenous populations or their lands, and research on cultural artefacts. The questionnaire can also be requested at the journal’s discretion for any other submissions, even if these conditions are not met.  Please find more information on the policy and a link to download a blank copy of the questionnaire here: https://journals.plos.org/plosone/s/best-practices-in-research-reporting. Please upload a completed version of your questionnaire as Supporting Information when you resubmit your manuscript.

3. Please provide additional details regarding participant consent. In the ethics statement in the Methods and online submission information, please ensure that you have specified (1) whether consent was informed and (2) what type you obtained (for instance, written or verbal, and if verbal, how it was documented and witnessed). If your study included minors, state whether you obtained consent from parents or guardians. If the need for consent was waived by the ethics committee, please include this information.

If you are reporting a retrospective study of medical records or archived samples, please ensure that you have discussed whether all data were fully anonymized before you accessed them and/or whether the IRB or ethics committee waived the requirement for informed consent. If patients provided informed written consent to have data from their medical records used in research, please include this information.

4. Your ethics statement should only appear in the Methods section of your manuscript. If your ethics statement is written in any section besides the Methods, please delete it from any other section.

5. We note that your Data Availability Statement is currently as follows: All relevant data are within the manuscript and its Supporting Information files.

Please confirm at this time whether or not your submission contains all raw data required to replicate the results of your study. Authors must share the “minimal data set” for their submission. PLOS defines the minimal data set to consist of the data required to replicate all study findings reported in the article, as well as related metadata and methods (https://journals.plos.org/plosone/s/data-availability#loc-minimal-data-set-definition).

For example, authors should submit the following data:

- The values behind the means, standard deviations and other measures reported;

- The values used to build graphs;

- The points extracted from images for analysis.

Authors do not need to submit their entire data set if only a portion of the data was used in the reported study.

If your submission does not contain these data, please either upload them as Supporting Information files or deposit them to a stable, public repository and provide us with the relevant URLs, DOIs, or accession numbers. For a list of recommended repositories, please see https://journals.plos.org/plosone/s/recommended-repositories.

If there are ethical or legal restrictions on sharing a de-identified data set, please explain them in detail (e.g., data contain potentially sensitive information, data are owned by a third-party organization, etc.) and who has imposed them (e.g., an ethics committee). Please also provide contact information for a data access committee, ethics committee, or other institutional body to which data requests may be sent. If data are owned by a third party, please indicate how others may request data access.

6. Thank you for stating the following financial disclosure:

funded by the Deanship of Research and Graduate Studies at King Khalid University through small group research under grant number RGP 1/4/46

Please state what role the funders took in the study. If the funders had no role, please state: "The funders had no role in study design, data collection and analysis, decision to publish, or preparation of the manuscript."

If this statement is not correct you must amend it as needed.

Please include this amended Role of Funder statement in your cover letter; we will change the online submission form on your behalf.

7. Thank you for stating the following in the Acknowledgments Section of your manuscript:

The authors extend their appreciation to the Deanship of Research and Graduate Studies at King Khalid University for funding this work through Small Group Research under grant number RGP 1/4/46.

We note that you have provided funding information that is not currently declared in your Funding Statement. However, funding information should not appear in the Acknowledgments section or other areas of your manuscript. We will only publish funding information present in the Funding Statement section of the online submission form.

Please remove any funding-related text from the manuscript and let us know how you would like to update your Funding Statement. Currently, your Funding Statement reads as follows:

funded by the Deanship of Research and Graduate Studies at King Khalid University through small group research under grant number RGP 1/4/46

Please include your amended statements within your cover letter; we will change the online submission form on your behalf.

8. Please amend your authorship list in your manuscript file to include author Rehab Mahmoud Abd El-Baky, Zeinab Fathalla, Hala Rady Ahmed, Ramadan Yahia, Mohamed A. Mawhoup, Ghada M. Sadiq, Hebatallah S. Barakat, Adel Al Fatease, Ali H. Alamri, Hamdy Abdelkader,

9. We note that Figure(s) 9, 10, 12, 14, 16 in your submission contain copyrighted images. All PLOS content is published under the Creative Commons Attribution License (CC BY 4.0), which means that the manuscript, images, and Supporting Information files will be freely available online, and any third party is permitted to access, download, copy, distribute, and use these materials in any way, even commercially, with proper attribution. For more information, see our copyright guidelines: http://journals.plos.org/plosone/s/licenses-and-copyright.

We require you to either (1) present written permission from the copyright holder to publish these figures specifically under the CC BY 4.0 license, or (2) remove the figures from your submission:

a. You may seek permission from the original copyright holder of Figure(s) 9, 10, 12, 14, 16 to publish the content specifically under the CC BY 4.0 license.

We recommend that you contact the original copyright holder with the Content Permission Form (http://journals.plos.org/plosone/s/file?id=7c09/content-permission-form.pdf) and the following text:

“I request permission for the open-access journal PLOS ONE to publish XXX under the Creative Commons Attribution License (CCAL) CC BY 4.0 (http://creativecommons.org/licenses/by/4.0/). Please be aware that this license allows unrestricted use and distribution, even commercially, by third parties. Please reply and provide explicit written permission to publish XXX under a CC BY license and complete the attached form.”

Please upload the completed Content Permission Form or other proof of granted permissions as an "Other" file with your submission.

In the figure caption of the copyrighted figure, please include the following text: “Reprinted from [ref] under a CC BY license, with permission from [name of publisher], original copyright [original copyright year].”

b. If you are unable to obtain permission from the original copyright holder to publish these figures under the CC BY 4.0 license or if the copyright holder’s requirements are incompatible with the CC BY 4.0 license, please either i) remove the figure or ii) supply a replacement figure that complies with the CC BY 4.0 license. Please check copyright information on all replacement figures and update the figure caption with source information. If applicable, please specify in the figure caption text when a figure is similar but not identical to the original image and is therefore for illustrative purposes only.

10. If the reviewer comments include a recommendation to cite specific previously published works, please review and evaluate these publications to determine whether they are relevant and should be cited. There is no requirement to cite these works unless the editor has indicated otherwise.

Additional Editor Comments :

Two reviewers assessed your manuscript and recommended substantial revisions

[Note: HTML markup is below. Please do not edit.]

Reviewers' comments:

Reviewer's Responses to Questions

**Comments to the Author**

1. Is the manuscript technically sound, and do the data support the conclusions?

The manuscript must describe a technically sound piece of scientific research with data that supports the conclusions. Experiments must have been conducted rigorously, with appropriate controls, replication, and sample sizes. The conclusions must be drawn appropriately based on the data presented.

Reviewer #1: Yes

Reviewer #2: Partly

**********

2. Has the statistical analysis been performed appropriately and rigorously?

Reviewer #1: Yes

Reviewer #2: I Don't Know

**********

3. Have the authors made all data underlying the findings in their manuscript fully available?

The PLOS Data policy requires authors to make all data underlying the findings described in their manuscript fully available without restriction, with rare exception (please refer to the Data Availability Statement in the manuscript PDF file). The data should be provided as part of the manuscript or its supporting information, or deposited to a public repository. For example, in addition to summary statistics, the data points behind means, medians and variance measures should be available. If there are restrictions on publicly sharing data—e.g. participant privacy or use of data from a third party—those must be specified.

Reviewer #1: Yes

Reviewer #2: Yes

**********

4. Is the manuscript presented in an intelligible fashion and written in standard English?

PLOS ONE does not copyedit accepted manuscripts, so the language in submitted articles must be clear, correct, and unambiguous. Any typographical or grammatical errors should be corrected at revision, so please note any specific errors here.

Reviewer #1: Yes

Reviewer #2: No

**********

5. Review Comments to the Author

Please use the space provided to explain your answers to the questions above. You may also include additional comments for the author, including concerns about dual publication, research ethics, or publication ethics. (Please upload your review as an attachment if it exceeds 20,000 characters)

Reviewer #1: The manuscript is well written and organized. Figures are well demonstrated with good reflection. Statistical results are mentioned properly. Pharmacokinetic parameters are not given. You can add PK study in this paper.

Reviewer #2: The study "Cyclodextrin Inclusion Complexes Enhance the Solubility and Anti-Virulence Activity of Metronidazole Against Uropathogenic Proteus mirabilisis" is methodologically sound and internally consistent, and the experimental dataset is substantial. However, over‑interpretation, conceptual ambiguity, and insufficient critical depth currently limit its suitability for publication without major revision. follwing are the major concerns and recommendations for authors.

Major Scientific Concerns

1. Anti‑virulence vs. Antibacterial Activity

The manuscript repeatedly alternates between antibacterial efficacy and anti‑virulence activity without clearly delineating the two concepts.

- MIC reduction is used as evidence of improved “anti‑virulence” activity, yet MIC is a growth inhibition metric, not a virulence‑specific endpoint.

- True anti‑virulence strategies are typically defined by effects at sub‑MIC levels without affecting growth. While sub‑MIC experiments are mentioned later (motility, urease, biofilm), the manuscript does not consistently frame or interpret results within this paradigm.

2. Over‑Interpretation of Molecular Docking Results

- Docking scores of −4 to −5 kcal/mol for CD inclusion are modest and fall within a range where enthalpy–entropy compensation and solvent effects dominate. Claiming “perfect fit” or “strongest interactions” is overstated.

- The assertion that metronidazole’s nitro group chelates both Ni²⁺ ions in urease similarly to acetohydroxamic acid is highly speculative. Nitroimidazoles are not established urease chelators in vivo.

- No experimental urease kinetics (IC₅₀, mode of inhibition) are provided to validate docking predictions.

3. Misinterpretation of FTIR and DSC as Proof of Inclusion

FTIR peak “weakening/disappearance” and DSC melting point suppression are repeatedly described as proof of complete inclusion complexation.

- FTIR spectral overlap with cyclodextrins frequently masks drug peaks even in simple physical mixtures.

- DSC melting peak disappearance can result from amorphization or dilution, not necessarily inclusion.

Authors can support these results with complementary techniques (PXRD, phase solubility diagrams, Job’s plot, NMR ROESY).

4. Dissolution Methodology and Interpretation

The “in vitro dissolution” experiment uses a Franz diffusion cell with a dialysis membrane, which measures diffusion + dissolution, not classical dissolution.

This setup is more representative of permeation release than intrinsic solubility.

- Interpretation as dissolution enhancement alone is therefore misleading.

Rename this experiment to “in vitro release/diffusion study” and clarify its limitations. Consider acknowledging that CDs may alter membrane transport rather than dissolution alone.

5. MIC Values Remain Extremely High

Even after formulation, MIC values remain in the mg/mL range (5–20 mg/mL), which is:

- Orders of magnitude higher than clinically relevant antibiotic MICs

- Not discussed critically in terms of translational relevance

Claiming “significant enhancement” without contextualizing these absolute values risks overstatement.

Discuss clinical relevance candidly. Emphasize anti‑virulence potential at sub‑MIC levels rather than antimicrobial potency.

6. Urease Assay Interpretation

The Christensen’s urea agar assay measures phenotypic urease activity, not direct enzyme inhibition.

- Reduced color change may reflect growth suppression, altered metabolism, or diffusion limitations, not enzyme inhibition.

- Yet results are discussed as “urease enzyme inhibition.”

Rephrase throughout to “suppression of urease activity/expression” rather than enzyme inhibition, unless biochemical assays are added.

Missing ares, author need to add out come of these

1. Phase solubility theory (AL/AN/AP types)– fundamental to CD inclusion science.

2. Comparative discussion of CD derivatives beyond HP‑β‑CD (e.g., SBE‑β‑CD, RM‑β‑CD).

3. Limitations of cyclodextrins (cholesterol extraction, membrane toxicity at high concentrations).

4. Recent anti‑virulence frameworks (QS inhibition vs metabolic interference).

5. In vivo relevance– no discussion of urinary dilution, urine composition, or catheter flow dynamics.

- Results are restated rather than critically analyzed.

- Contradictions are not explored (e.g., α‑CD showing activity despite poor inclusion).

- No comparison is made with alternative solubility enhancers or non‑CD systems.

- Why HP‑β‑CD outperforms β‑CD beyond solubility (steric shielding, hydration shell).

- Whether observed anti‑virulence effects stem from CDs themselves.

What are the limitations of docking‑driven mechanistic ?.

Data Presentation and Interpretation

- Tables lack statistical annotations.

- MIC table lacks confidence intervals or replicate variance.

- Molecular docking content is duplicated across sections.

Condense docking description into a single cohesive subsection and tighten the Discussion.

Professional language editing is strongly advised.

Limitations

- Conceptual novelty is incremental, not transformative.

- No new formulation principle or mechanistic model is proposed.

Specific Recommendations for Improvement

1. Rewrite Introduction to clearly define anti‑virulence vs antibacterial activity.

2. Soften mechanistic claims from docking and FTIR/DSC.

3. Clarify dissolution vs diffusion methodology.

4. Reframe urease results as phenotypic suppression.

5. Add a limitations subsection in Discussion.

6. Tighten language and reduce redundancy.

7. Consider removing or shortening repetitive docking descriptions.

**********

6. PLOS authors have the option to publish the peer review history of their article (what does this mean?). If published, this will include your full peer review and any attached files.

If you choose “no”, your identity will remain anonymous but your review may still be made public.

**Do you want your identity to be public for this peer review?**  For information about this choice, including consent withdrawal, please see our Privacy Policy.

Reviewer #1: No

Reviewer #2: No

**********

[NOTE: If reviewer comments were submitted as an attachment file, they will be attached to this email and accessible via the submission site. Please log into your account, locate the manuscript record, and check for the action link "View Attachments". If this link does not appear, there are no attachment files.]

To ensure your figures meet our technical requirements, please review our figure guidelines: https://journals.plos.org/plosone/s/figures

You may also use PLOS’s free figure tool, NAAS, to help you prepare publication quality figures: https://journals.plos.org/plosone/s/figures#loc-tools-for-figure-preparation.

NAAS will assess whether your figures meet our technical requirements by comparing each figure against our figure specifications.

# Author comment: Author response to Decision Letter 1

**DOI:** 10.1371/journal.pone.0353058.r002

12 May 2026

Dear Editor,

We sincerely thank you and the reviewers for the thorough evaluation of our manuscript entitled:

“Cyclodextrin Inclusion Complexes Enhance the Solubility and Anti-Virulence Activity of Metronidazole Against Uropathogenic Proteus mirabilis.”

We have carefully revised the manuscript in response to all reviewers' and editors' comments. Major revisions include:

• Clear conceptual distinction between antibacterial and anti-virulence activity throughout the manuscript.

• Substantial reduction of mechanistic overstatement regarding molecular docking and urease interaction.

• Reframing urease findings as suppression of urease-associated phenotype rather than confirmed enzymatic inhibition.

• Clarification that the in vitro Franz diffusion system reflects release/diffusion behavior rather than intrinsic dissolution alone.

• Addition of a dedicated “Limitations of the Study” subsection.

• Contextual discussion of absolute MIC values and translational relevance.

• Condensation and tightening of the molecular docking sections.

• Professional English language editing throughout.

________________________________________

Ethics Statement Clarification

The study involved the secondary use of a clinical bacterial isolate obtained from laboratory diagnostics. No patient identifiers were accessed. The Ethics Committee of the Faculty of Pharmacy, Deraya University (Approval No. 8/2024) reviewed the protocol and waived the requirement for informed consent, as the study involved anonymized microbial samples without direct or indirect patient contact.

The ethics statement now appears only in the Methods section, in accordance with journal requirements.

________________________________________

Funding Statement Amendment

The Funding Statement has been amended to read:

“This work was funded by the Deanship of Research and Graduate Studies at King Khalid University through Small Group Research under grant number RGP 1/4/46. The funders had no role in study design, data collection and analysis, decision to publish, or preparation of the manuscript.”

It is a contractual obligation to acknowledge the Funding information

________________________________________

Data Availability Confirmation

All raw data underlying the findings, including replicate values used for statistical analysis and figure generation, are provided in the Supporting Information files. This includes raw values for MIC assays, motility measurements, urease-associated phenotype assays, biofilm quantification, and release/diffusion studies.

________________________________________

Authorship

The authorship list has been updated to include all listed authors as requested.

________________________________________

Figures

All figures are original and were generated by the authors. No copyrighted third-party material has been used. For example: these plates are from those in the manuscript before cropping edges

________________________________________

We believe the revised manuscript now addresses all scientific, conceptual, and editorial concerns raised during peer review. We sincerely appreciate the opportunity to improve our work and look forward to your further consideration.

Thank you for your time and careful evaluation.

Sincerely,

Prof. Rehab Mahmoud Abd El-Baky

Corresponding Author

Faculty of Pharmacy

Minia University & Deraya University

Email: rehab.mahmoud@mu.edu.eg

Point to point reply

Reviewer comments Author reply

Reviewer #1:

The manuscript is well written and organized. Figures are well demonstrated with good reflection.

Statistical results are mentioned properly.

Pharmacokinetic parameters are not given. You can add PK study in this paper We thank the reviewer for the encouraging comments and valuable suggestions.

The present study was designed as an in vitro pharmaceutical and anti-virulence investigation, focusing on cyclodextrin complexation, physicochemical characterization, release behavior, and phenotypic modulation of virulence factors. Therefore, in vivo pharmacokinetic (PK) experiments were not included within the scope of the current work.

To address this point, we have now added a paragraph in the Discussion section (Limitations of the Study) acknowledging the absence of pharmacokinetic evaluation and highlighting it as an important direction for future research. Specifically, we state that future studies should evaluate pharmacokinetics and urinary drug concentrations in relevant in vivo infection models.

Reviewer #2: We sincerely thank the reviewer for the detailed and insightful critique. The manuscript has been substantially revised to address all conceptual, methodological, and interpretational concerns.

The study "Cyclodextrin Inclusion Complexes Enhance the Solubility and Anti-Virulence Activity of Metronidazole Against Uropathogenic Proteus mirabilisis" is methodologically sound and internally consistent, and the experimental dataset is substantial. However, over interpretation, conceptual ambiguity, and insufficient critical depth currently limit its suitability for publication without major revision. Following are the major concerns and recommendations for authors.

Reviewer 2: Major Scientific Concerns:

1. Anti virulence vs. Antibacterial Activity

The manuscript repeatedly alternates between antibacterial efficacy and anti virulence activity without clearly delineating the two concepts.

- MIC reduction is used as evidence of improved “anti virulence” activity, yet MIC is a growth inhibition metric, not a virulence specific endpoint.

- True anti virulence strategies are typically defined by effects at sub MIC levels without affecting growth. While sub MIC experiments are mentioned later (motility, urease, biofilm), the manuscript does not consistently frame or interpret results within this paradigm. We agree with the reviewer that the conceptual distinction between antibacterial activity and anti-virulence modulation required clearer presentation.

The manuscript has been revised as follows:

• The Introduction section has been rewritten to clearly define anti-virulence strategies.

• A clear distinction between MIC (growth inhibition) and sub-MIC anti-virulence endpoints has been introduced.

• MIC reduction is now interpreted as improved antimicrobial exposure resulting from cyclodextrin complexation, rather than direct anti-virulence activity.

• Motility, urease-associated phenotype, and biofilm assays are now explicitly framed as sub-MIC anti-virulence evaluations.

These changes improve conceptual clarity throughout the manuscript.

2. Over Interpretation of Molecular Docking Results

- Docking scores of −4 to −5 kcal/mol for CD inclusion are modest and fall within a range where enthalpy–entropy compensation and solvent effects dominate. Claiming “perfect fit” or “strongest interactions” is overstated.

- The assertion that metronidazole’s nitro group chelates both Ni²⁺ ions in urease similarly to acetohydroxamic acid is highly speculative. Nitroimidazoles are not established urease chelators in vivo.

- No experimental urease kinetics (IC₅₀, mode of inhibition) are provided to validate docking predictions.

We appreciate this important observation.

The molecular docking section has been carefully revised to avoid over-interpretation:

• Statements such as “perfect fit” and “strongest interactions” have been removed.

• Docking scores (−4 to −5 kcal/mol) are now described as indicative of possible compatibility between metronidazole and cyclodextrin cavities rather than strong binding interactions.

• Claims suggesting Ni²⁺ chelation within the urease active site have been removed.

• A paragraph discussing limitations of molecular docking, including solvent effects and enthalpy–entropy compensation, has been added.

• Docking findings are now described as predictive and hypothesis-generating rather than mechanistically confirmatory.

Additionally, duplicated docking descriptions were condensed into a single subsection.

1. Misinterpretation of FTIR and DSC as Proof of Inclusion

FTIR peak “weakening/disappearance” and DSC melting point suppression are repeatedly described as proof of complete inclusion complexation.

- FTIR spectral overlap with cyclodextrins frequently masks drug peaks even in simple physical mixtures.

- DSC melting peak disappearance can result from amorphization or dilution, not necessarily inclusion.

Authors can support these results with complementary techniques (PXRD, phase solubility diagrams, Job’s plot, NMR ROESY).

We agree.

We have:

• Removed statements claiming “proof of complete inclusion”.

• Replaced them with “consistent with possible inclusion complex formation”.

• Added a paragraph acknowledging that PXRD, ROESY-NMR, or phase solubility studies would provide stronger evidence.

• Added this as a limitation of the current work.

2. Dissolution Methodology and Interpretation

The “in vitro dissolution” experiment uses a Franz diffusion cell with a dialysis membrane, which measures diffusion + dissolution, not classical dissolution.

This setup is more representative of permeation release than intrinsic solubility.

- Interpretation as dissolution enhancement alone is therefore misleading.

Rename this experiment to “in vitro release/diffusion study” and clarify its limitations. Consider acknowledging that CDs may alter membrane transport rather than dissolution alone.

Correct.

We have:

• Renamed the section to “In vitro release/diffusion study”.

• Clarified the experimental setup.

• Explained that results reflect release across a membrane rather than intrinsic solubility alone.

• Added a limitations statement.

3. MIC Values Remain Extremely High

Even after formulation, MIC values remain in the mg/mL range (5–20 mg/mL), which is:

- Orders of magnitude higher than clinically relevant antibiotic MICs

- Not discussed critically in terms of translational relevance

Claiming “significant enhancement” without contextualizing these absolute values risks overstatement.

Discuss clinical relevance candidly. Emphasize anti virulence potential at sub MIC levels rather than antimicrobial potency.

We agree with the reviewer and have revised the Discussion accordingly.

The manuscript now explicitly acknowledges that the observed MIC values remain within the mg/mL range, which is substantially higher than those typically reported for clinically potent antibiotics.

Therefore, the study is now clearly framed within an anti-virulence modulation context, emphasizing that the most relevant findings relate to phenotypic modulation observed at sub-MIC concentrations, including:

• motility reduction

• suppression of urease-associated phenotype

• inhibition of biofilm formation

These endpoints represent virulence modulation rather than bactericidal activity.

4. Urease Assay Interpretation

The Christensen’s urea agar assay measures phenotypic urease activity, not direct enzyme inhibition.

- Reduced color change may reflect growth suppression, altered metabolism, or diffusion limitations, not enzyme inhibition.

- Yet results are discussed as “urease enzyme inhibition.”

Rephrase throughout to “suppression of urease activity/expression” rather than enzyme inhibition, unless biochemical assays are added.

We agree with the reviewer.

The manuscript has been revised as follows:

• The term “urease inhibition” has been replaced with “suppression of urease-associated phenotype.”

• The text now clarifies that Christensen’s urea agar measures phenotypic urease activity under growth conditions rather than direct enzymatic inhibition.

• Statements implying direct catalytic inhibition have been removed.

5. Missing areas, author need to add out come of these

1. Phase solubility theory (AL/AN/AP types)– fundamental to CD inclusion science.

2. Comparative discussion of CD derivatives beyond HP β CD (e.g., SBE β CD, RM β CD).

3. Limitations of cyclodextrins (cholesterol extraction, membrane toxicity at high concentrations).

4. Recent anti virulence frameworks (QS inhibition vs metabolic interference).

5. In vivo relevance– no discussion of urinary dilution, urine composition, or catheter flow dynamics.

6. Results are restated rather than critically analyzed.

- Contradictions are not explored (e.g., α CD showing activity despite poor inclusion).

- No comparison is made with alternative solubility enhancers or non CD systems.

- Why HP β CD outperforms β CD beyond solubility (steric shielding, hydration shell).

- Whether observed anti virulence effects stem from CDs themselves.

What are the limitations of docking driven mechanistic ?.

7. Data Presentation and Interpretation

- Molecular docking content is duplicated across sections.

Condense docking description into a single cohesive subsection and tighten the Discussion.

8. Professional language editing is strongly advised.

9. Limitations

- Conceptual novelty is incremental, not transformative.

- No new formulation principle or mechanistic model is proposed.

Specific Recommendations for Improvement

1. Rewrite Introduction to clearly define anti virulence vs antibacterial activity.

2. Soften mechanistic claims from docking and FTIR/DSC.

3. Clarify dissolution vs diffusion methodology.

4. Reframe urease results as phenotypic suppression.

5. Add a limitations subsection in Discussion.

6. Tighten language and reduce redundancy.

7. Consider removing or shortening repetitive docking descriptions.

________________________________________ We thank the reviewer for this helpful suggestion.

• Phase solubility theory based on the Higuchi–Connors model (AL-type systems) extensibvely studied and the main foculs of this manuscript is to study the effect of cyclodextrins pn the antibacterial activities of metronidazole but not solubility.

• Cyclodextrin is regarded as safe excipients but the issue of cholesterol extraction if applied on mucous membrane such as eye or injected and the toxicity of these excipients is beyond the current investigation.

• Comparison with alternative cyclodextrin derivatives, including SBE-β-CD and RM-β-CD

• Potential limitations of cyclodextrins, including membrane cholesterol extraction and concentration-dependent toxicity

• Integration of anti-virulence conceptual frameworks, including phenotypic modulation and metabolic interference mechanisms

These additions strengthen the theoretical context of the study.

The manuscript has undergone professional language editing to improve clarity, grammar, and overall readability.

We agree that the study represents an incremental but meaningful formulation-based investigation.

To address this point, a dedicated “Limitations of the Study” subsection has been added in the Discussion, outlining:

• absence of phase solubility experiments

• lack of enzyme kinetics validation

• in vitro experimental design

• need for future in vivo pharmacokinetic and infection model studies

We sincerely thank the reviewers for his recommendations and comments, which significantly improved the clarity, balance, and scientific rigor of our manuscript.

All recommendations were taken in consideration in response and modifying our manuscript

Attachment Submitted filename: Authors reply.docx

# Editor report: Decision Letter 1

**DOI:** 10.1371/journal.pone.0353058.r003

**Authors:** Reham Mokhtar ELTarabili

18 Jun 2026

Cyclodextrin Inclusion Complexes Enhance the Solubility and Anti-Virulence Activity of Metronidazole Against Uropathogenic Proteus mirabilis

PONE-D-25-60159R1

Dear Dr. Abd El-Baky,

We’re pleased to inform you that your manuscript has been judged scientifically suitable for publication and will be formally accepted for publication once it meets all outstanding technical requirements.

Within one week, you’ll receive an e-mail detailing the required amendments. When these have been addressed, you’ll receive a formal acceptance letter and your manuscript will be scheduled for publication.

An invoice will be generated when your article is formally accepted. Please note, if your institution has a publishing partnership with PLOS and your article meets the relevant criteria, all or part of your publication costs will be covered. Please make sure your user information is up-to-date by logging into Editorial Manager at Editorial Manager® and clicking the ‘Update My Information' link at the top of the page. For questions related to billing, please contact billing support.

If your institution or institutions have a press office, please notify them about your upcoming paper to help maximize its impact. If they’ll be preparing press materials, please inform our press team as soon as possible -- no later than 48 hours after receiving the formal acceptance. Your manuscript will remain under strict press embargo until 2 pm Eastern Time on the date of publication. For more information, please contact onepress@plos.org.

Kind regards,

Reham Mokhtar ELTarabili

Academic Editor

PLOS One

Additional Editor Comments (optional):

Reviewers' comments:

# Editor report: Acceptance letter

**DOI:** 10.1371/journal.pone.0353058.r004

**Authors:** Reham Mokhtar ELTarabili

PONE-D-25-60159R1

PLOS One

Dear Dr. Abd El-Baky,

I'm pleased to inform you that your manuscript has been deemed suitable for publication in PLOS One. Congratulations! Your manuscript is now being handed over to our production team.

At this stage, our production department will prepare your paper for publication. This includes ensuring the following:

* All references, tables, and figures are properly cited

* All relevant supporting information is included in the manuscript submission,

* There are no issues that prevent the paper from being properly typeset

You will receive further instructions from the production team, including instructions on how to review your proof when it is ready. Please keep in mind that we are working through a large volume of accepted articles, so please give us a few days to review your paper and let you know the next and final steps.

Lastly, if your institution or institutions have a press office, please let them know about your upcoming paper now to help maximize its impact. If they'll be preparing press materials, please inform our press team within the next 48 hours. Your manuscript will remain under strict press embargo until 2 pm Eastern Time on the date of publication. For more information, please contact onepress@plos.org.

You will receive an invoice from PLOS for your publication fee after your manuscript has reached the completed accept phase. If you receive an email requesting payment before acceptance or for any other service, this may be a phishing scheme. Learn how to identify phishing emails and protect your accounts at https://explore.plos.org/phishing.

If we can help with anything else, please email us at customercare@plos.org.

Thank you for submitting your work to PLOS ONE and supporting open access.

Kind regards,

PLOS ONE Editorial Office Staff

on behalf of

Dr. Reham Mokhtar ELTarabili

Academic Editor

PLOS One
